# Distinct immune signatures define hepatitis D– versus hepatitis B–associated hepatocellular carcinoma

**DOI:** 10.1097/HC9.0000000000001060

**Published:** 2026-10-19

**Authors:** Simon Glimmann, Katja Deterding, Kilian Bock, Birgit Bremer, Anke R.M. Kraft, Markus Cornberg, Heiner Wedemeyer, Bernd Heinrich, Anika Wranke

**Affiliations:** 1 Department of Gastroenterology, Hepatology, Infectious Diseases and Endocrinology, Hannover Medical School, Hannover, Germany; 2 German Centre for Infection Research (DZIF), Partner Site Hannover-Braunschweig, Hannover, Germany; 3 Centre for Individualised Infection Medicine (CiiM), a joint venture between the Helmholtz Centre for Infection Research (HZI) and Hannover Medical School (MHH), Hannover, Germany; 4 Cluster of Excellence RESIST (EXC 2155), Hannover Medical School, Hannover, Germany; 5 D-SOLVE, EU-funded Network on individualized management of hepatitis D, Hannover, Germany

**Keywords:** hepatitis B virus, hepatitis D virus, hepatocellular carcinoma, immune dysregulation, proximity extension assay, soluble inflammatory mediators

## Abstract

**Background:**

Hepatocellular carcinoma (HCC) is a leading cause of cancer-related mortality worldwide. Hepatitis D is the most severe form of viral hepatitis. However, the clinical, virological, and systemic inflammatory characteristics of HDV-associated HCC remain insufficiently characterized.

**Methods:**

In this single-center retrospective study, we identified 162 patients with HCC due to chronic hepatitis D (HDV-HCC; n=54) or chronic hepatitis B (HBV-HCC; n=108). Plasma levels of 92 soluble inflammatory mediators (SIMs) were quantified using proximity extension assay in HCC patients [HBV-HCC (n=50), HDV-HCC (n=30), and matched non-HCC controls (HBV=19, HDV=17)].

**Results:**

At the time of HCC diagnosis, patients with CHD were younger, had more impaired liver synthesis and detoxification function, and lower levels of AST, CRP, and leukocytes. HDV-HCCs presented with smaller tumors, less frequent portal vein infiltration, and more often fulfilled Milan criteria (all *p*<0.01). Systemic immune profiling revealed distinct inflammatory patterns between both etiologies. Compared with HBV-HCC, HDV-HCC was characterized by a comparatively stronger IL-10–associated immunoregulatory signature, whereas HBV-HCC exhibited a more pronounced pro-inflammatory profile involving IL-6– and phosphorylation- and proliferation–associated signaling. Despite these differences, event-free survival did not differ between groups.

**Conclusions:**

HBV-HCC more frequently presented with advanced tumor stage, whereas HDV-HCC was associated with more severe underlying liver disease but smaller tumors. Importantly, HBV-associated and HDV-associated HCC exhibit distinct systemic inflammatory signatures, potentially reflecting differences in systemic immune regulation between etiologies. These findings may have implications for biomarker development and future etiology-specific therapeutic strategies.

## INTRODUCTION

HDV requires hepatitis B surface antigen (HBsAg) for viral assembly and can therefore infect only individuals with concurrent HBV infection or by superinfection in chronic HBsAg carriers.^1^ It is estimated that ~15–20 million HBsAg carriers are coinfected with HDV worldwide.^2^ Hepatitis delta is the most severe form of viral hepatitis, leading to high rates of liver-related decompensation, liver transplantation, and death.^3^,^4^,^5^ HCC is one of the most common cancers and a leading cause of cancer-related mortality worldwide.^6^ HCC usually occurs in the setting of underlying liver disease, with cirrhosis being present in ~80%–90% of cases. Whether HDV infection itself contributes to hepatocarcinogenesis or whether the increased risk is driven by accelerated progression to cirrhosis remains debated;^7^,^8^,^9^ however, multiple pooled analyses have demonstrated an increased risk of HCC among patients with chronic hepatitis D (CHD) compared with chronic hepatitis B (CHB) patients.^8^,^9^ Nevertheless, it remains unclear whether HDV-associated HCC differs from HBV-associated HCC in terms of tumor biology and clinical course. Previous comparative studies have primarily focused on clinical characteristics, which suggested that HDV-associated HCC may predominantly arise in the context of advanced underlying liver disease.^10^,^11^ In contrast, Huo et al^12^ reported largely similar clinical characteristics and no significant differences in survival between HBV-associated and HDV-associated HCC. Interestingly, HDV-HCC patients have been reported to present with smaller tumors despite more advanced liver disease, suggesting distinct mechanisms of hepatocarcinogenesis,^11^ although whether these are driven by direct oncogenic effects of HDV or by accelerated progression to cirrhosis remains debated.^13^ In addition, adaptive and innate immunological mechanisms may play an important role in the development and clinical course of HCC caused by HDV, in contrast to HBV alone. Studies in non-cancer HDV coinfected patients have demonstrated enhanced activation of the innate immune system, including increased NK-cell frequencies, although their functional capacity remains unclear. These findings suggest a dysregulated immune environment that may contribute to hepatocarcinogenesis in HDV infection.^14^,^15^ However, despite these observations, the immunological landscape associated with HDV-associated hepatocarcinogenesis remains poorly understood. In particular, it is unclear whether HDV-HCC is characterized by distinct systemic inflammatory signatures compared with HBV-HCC and how these signatures relate to clinical and tumor-associated characteristics. Therefore, the aim of this study was to compare clinical characteristics and systemic inflammatory mediator profiles between HDV-HCC and HBV-HCC and to identify inflammatory pathways associated with etiology-specific HCC.

## METHODS

### Study population and design

Patients with evidence of HBV infection (HBeAg, HBsAg, HBV DNA) and/or HDV infection (anti-HDV, HDV RNA) were selected from a database of 725 patients followed at Hannover Medical School between 1990 and 2023. Patients were eligible for inclusion if they had a confirmed diagnosis of hepatocellular carcinoma (HCC) and at least 1 follow-up visit. A retrospective analysis revealed 108 HBV-HCC and 54 HDV-HCC cases. Cirrhosis was diagnosed by histology (Ishak F5–F6), transient elastography (>14 kPa), or imaging evidence of nodular liver morphology with signs of portal hypertension. Liver function was assessed using MELD and Child–Pugh scores. Performance status was evaluated using the ECOG score. HCC diagnosis was established either histologically or based on radiological criteria according to current clinical guidelines, requiring characteristic features on at least 2 imaging modalities.^16^ Tumor characteristics included size, number, and growth pattern. Tumor stage was classified according to the Milan criteria.^17^ Tumor-directed therapies were recorded longitudinally throughout follow-up. Histological tumor grade (G1–G4) was assessed as described previously.^18^

### SIM quantification

To characterize the systemic inflammatory milieu at the time of HCC diagnosis and before tumor-directed therapy, patients were screened for the availability of baseline plasma samples. Baseline plasma samples were available from 88 patients (HBV-HCC n=53; HDV-HCC n=35). A matched control cohort without HCC was established (HBV n=19; HDV n=17), matched for age, sex, HBeAg status, INR, bilirubin, and nucleos(t)ide analogue (NA) therapy. Control patients had no history of hepatic decompensation or HCC during follow-up. Plasma samples were stored at −20 °C, aliquoted (50 μL EDTA plasma), and analyzed using a proximity extension assay (PEA; Olink Proteomics, Uppsala, Sweden). A predefined inflammation panel (Target 96) enabling quantification of 92 soluble immune mediators (SIMs) was applied (Supplemental Table S1, https://links.lww.com/HC9/C506). Data were normalized using internal controls and reported as log2-transformed normalized protein expression (NPX) values. Markers failing quality control or below the limit of detection were excluded from further analysis. After quality control, 78 SIMs were retained for analysis across 50 HBV-HCC, 30 HDV-HCC, and 36 non-HCC control samples.

### Statistics

Statistical analyses were performed using SPSS software (version 28; IBM Corp., Chicago, IL, USA). Continuous variables are reported as mean±SD. A two-sided *p*-value <0.05 was considered statistically significant. Continuous variables were compared using the Student *t* test for normally distributed data and the Mann–Whitney *U* test for non-parametric data. Categorical variables were compared using the χ^2^ test or the Fisher exact test, as appropriate. The Spearman rank correlation was used for correlation analyses. To account for multiple testing, *p*-values were corrected using the Benjamini–Hochberg false discovery rate (FDR) method. FDR-significant mediators were subsequently assessed in multivariable linear regression analyses adjusted for the Model for End-Stage Liver Disease (MELD) score to account for liver disease severity. Soluble immune mediator concentrations were modeled as dependent variables with HCC status or viral etiology as predictors. In addition, sensitivity analyses restricted to cirrhotic HCC patients were performed to evaluate the robustness of the main findings. Graphical representations were generated using GraphPad Prism (version 10; GraphPad Software, San Diego, CA, USA). Heatmaps were generated based on Spearman correlation matrices. FDR-corrected significantly differentially expressed SIMs were subjected to pathway enrichment and protein–protein interaction analyses using g:Profiler (June 2025) and STRING (version 12.0).

### Ethics statement

The study was approved by the Ethics Committee of Hannover Medical School (Hannover, Germany; approval no. 11028_BO_K_2023) and was conducted in accordance with the ethical principles of the Declarations of Helsinki and Istanbul. Written informed consent was obtained from all participants in accordance with institutional requirements.

## RESULTS

### Clinical characteristics

From a monocentric database, 54 patients with HDV-HCC and 108 patients with HBV-HCC were initially identified (Figures 1A, B).

**FIGURE 1:**
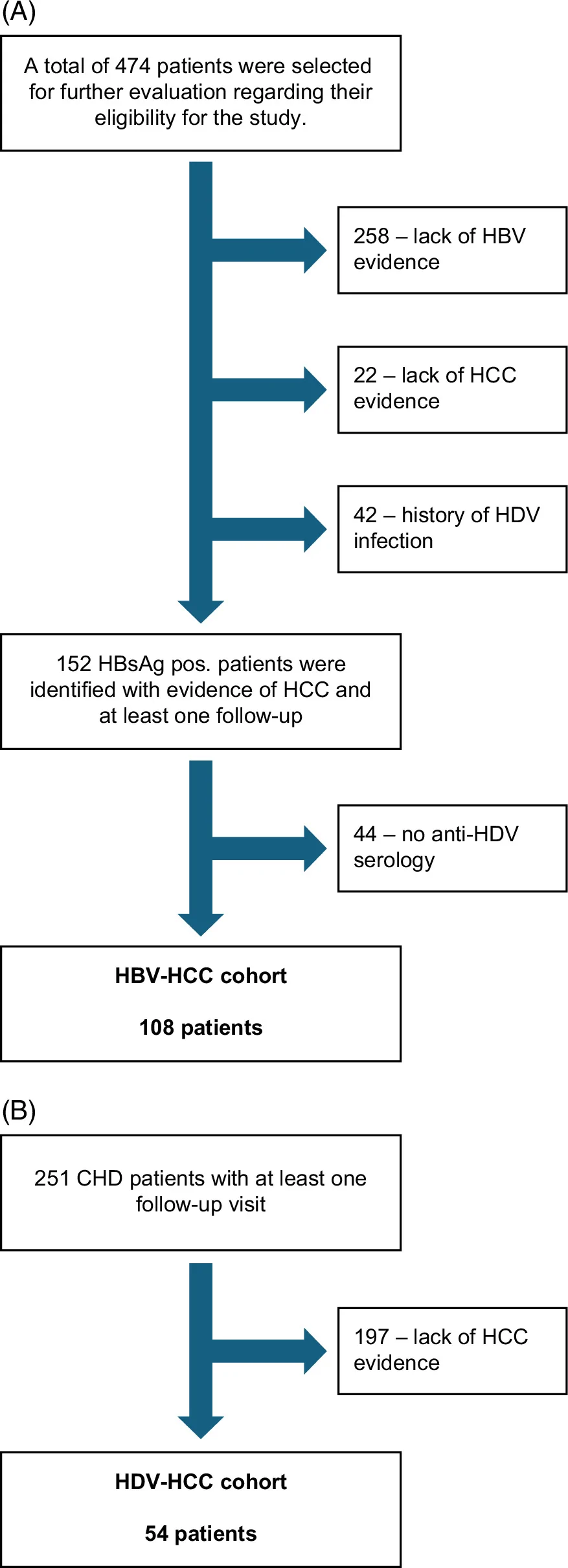
(A) Flowchart of patient selection for the HBV cohort. (B) Flowchart of patient selection for the HDV cohort. Abbreviations: CHD, chronic hepatitis D; HBsAg pos., hepatitis B surface antigen positive; HBV, hepatitis B virus; HCC, hepatocellular carcinoma; HDV, hepatitis D virus​​​​​.

#### Clinical comparison of HBV-HCC and HDV-HCC

The comparison of patient characteristics is presented in Table 1A. Most patients were male (85%). Patients with HDV-HCC presented with more advanced liver dysfunction, reflected by higher INR, lower platelet counts and albumin levels, and higher bilirubin concentrations (all *p*≤0.04). In contrast, systemic inflammatory markers, including leukocyte count, CRP, and AST, were significantly higher in HBV-HCC compared with HDV-HCC (all *p*≤0.05). Patients with HDV-HCC were also found to be younger (mean age 51.3 vs. 59.1 years; *p*<0.01). HDV-HCC patients more frequently exhibited impaired performance status (ECOG ≥1) (*p*=0.04). Previous hepatic decompensation was more common in HDV-HCC (45.5% vs. 25.8%, *p*=0.02). Child–Pugh score, MELD score, AFP levels, and metabolic comorbidities did not differ between groups. Patients with HDV-HCC had evidence of liver cirrhosis in 92.6% of cases, compared with 81.5% in HBV-HCC. Notably, all HDV-HCC patients without histologically confirmed cirrhosis showed evidence of advanced fibrosis or indirect signs of cirrhosis.

**TABLE 1A:** Baseline demographic, clinical, virological, and laboratory characteristics of patients with HDV-associated and HBV-associated hepatocellular carcinoma at the time of HCC diagnosis

	HDV-HCC (n=54)	HBV-HCC (n=108)	*p*
Age (y), mean±SD (range)	51.3±7.8 (32.3–67.5)	59.1±11.6 (23.3–84.4)	<0.01
Male (%, n)	79.6% (43)	88.0% (95)	0.16
BMI (kg/m^2^), mean±SD (range)	26.5±3.2 (20.9–33.5)	27.2±4.6 (17.9–44.1)	0.40
Diabetes mellitus (%, n)	12.5% (6)	23.6% (25)	0.11
ECOG (%, n)			
0	70.0% (35)	88.0% (81)	
1	18.0% (9)	6.5% (6)	0.04
2	10.0% (5)	5.4% (5)	
3	2.0% (1)	0% (0)	
Smoker (%, n)	40.9% (18)	47.9% (34)	0.46
Alcohol abuse (%, n)	17.0% (8)	15.7% (11)	0.85
Substance abuse (%, n)			
None	52.2% (60)	52.1% (37)	0.77
Tabacco	45.2% (52)	47.9% (34)	
Others	6.8% (3)	0% (0)	
Liver comorbidities (%, n)			
None	80.4% (41)	89.7% (96)	
MASLD	5.9% (3)	1.9% (2)	0.37
MetALD	0% (0)	0% (0)	
ALD	9.8% (5)	6.5% (7)	
Others	3.9% (2)	1.9% (2)	
Steatosis (%, n)	88.9% (8)	100% (6)	1.0
Liver cirrhosis (%, n)	92.6% (50)	81.5% (88)	0.08
Child–Pugh score (%, n)			
A	46.0% (23)	62.4% (53)	0.07
B	40.0% (20)	32.9% (28)	
C	14.0% (7)	4.7% (4)	
Previous antiviral therapy (%, n)	74.5% (38)	56.3% (58)	0.03
Previous NA exposure (%, n)			
None	32.0% (16)	25.3% (19)	<0.01
NA	38.0% (19)	70.7% (53)	
NA + IFN	30.0% (15)	4.0% (3)	
NA + BLV	0% (0)	0% (0)	
Previous IFN exposure (%, n)	41.2% (21)	5.9% (3)	<0.01
Therapy at HCC diagnosis (%, n)	69.0% (35)	59.0% (61)	0.33
No therapy (n):	17	38	
Unknown (n):	2	5	
Esophageal varices (%, n)			
None	26.2% (11)	30.3% (20)	0.09
Grade 1	19.0% (8)	33.3% (22)	
Grade 2	19.0% (8)	22.7% (15)	
Grade 3	33.3% (14)	12.1% (8)	
Grade 4	2.4% (1)	1.5% (1)	
Previous decompensation (%, n)	45.5% (20)	25.8% (22)	0.02
HBsAg quantitative (IU/mL), mean±SD (range)	7821.39±7719.8 (0–20,000)	5073.3±12,902.9 (0–102,890)	0.22
HBV DNA quantitative (IU/mL), mean±SD (range)	1752.6±4467.9 (0–20,000)	19,557,632.3±140,875,930.9 (0–1,200,000,000)	0.41
HBV DNA positive (qualitative) (%, n)	64.9% (24)	78.1% (57)	0.14
HBeAg qualitative (%, n)	18.2% (6)	9.1% (8)	0.16
HDV RNA (IU/mL), mean±SD (range)	190,085±455,339.8 (600–2,100,000)		
HDV RNA positive (qualitative) (%, n)	65.9% (27/41)		
Creatinine (µmol/L), mean±SD (range)	79.3±28.2 (38–170)	89.3±40.6 (1.19–241)	0.12
AST (U/L), mean±SD (range)	94.22±57.7 (29–280)	141.95±225.8 (20–1473)	0.05
ALT (U/L), mean±SD (range)	74.2±45.9 (14–220)	73.2±102.9 (7–890)	0.95
Alcaline phosphatase (U/L), mean±SD (range)	138.9±70.2 (24–408)	159.3±135.8 (35–941)	0.33
Gamma-glutamyltransferase (U/L), mean±SD (range)	159.8±248.7 (17–1634)	206.5±229.4 (12–1052)	0.25
Cholinesterase (kU/L), mean±SD (range)	3.75±2.1 (0.8–9.0)	6.8±16.1 (0.5–162)	0.24
LDH (U/L), mean±SD (range)	247.8±98.9 (138.0–544.0)	248.0±99.0 (80.0–816.0)	0.98
Billirubin (mg/dL), mean±SD (range)	51.0±115.3 (6.0–652.0)	26.4±33.6 (3.0–255.0)	0.04
Total protein (g/L), mean±SD (range)	70.7±8.1 (48–84)	73.5±6.0 (57–95)	0.05
Albumin (g/L), mean±SD (range)	30.9±7.2 (16–44)	34.4±6.0 (18–47)	0.03
Leukocytes (10^3^/μL), mean±SD (range)	5.0±2.2 (0.7–11.8)	6.2±2.8 (1.8–18.6)	<0.01
Hemoglobin (g/dL), mean±SD (range)	12.4±2.5 (6.3–16.2)	13.2±1.7 (9–16.6)	0.37
Platelets (10^3^/μL), mean±SD (range)	105.0±51.3 (17–223)	163.1±109.7 (20–499)	<0.01
INR, mean±SD (range)	1.35±0.39 (0.96–3.14)	1.19±0.16 (0.9–1.82)	<0.01
AFP (U/L), mean±SD (range)	1038.9±3570.6 (2–14,535.0	8725.3± 32.6 (1.3–157,382.0)	0.06
CA 19-9 (U/mL), mean±SD (range)	41.7±33.3 (1–122)	160.6±788.3 (1–5600)	0.46
CEA (ng/mL), mean±SD (range)	4.7±3.2 (1–12)	3.8±4.4 (1–26)	0.43
CRP (mg/L), mean±SD (range)	7.84±8.0 (1–35)	22.6±36.5 (1–179)	<0.01
MELD score, mean±SD (range)	12.2±6.1 (6.3–37.8)	10.8±3.8 (6.0–21.3)	0.13

*Note:* Continuous variables are presented as mean±standard deviation (SD) with range. Categorical variables are presented as percentages (n). *p*-values <0.05 were considered statistically significant.

Abbreviations: AFP, alpha-fetoprotein; ALD, alcohol-associated liver disease; ALT, alanine aminotransferase; AST, aspartate aminotransferase; BCLC, Barcelona Clinic Liver Cancer; BLV, bulevirtide; BMI, body mass index; CA 19-9, carbohydrate antigen 19-9; CEA, carcinoembryonic antigen; CHB, chronic hepatitis B; CHD, chronic hepatitis D; CRP, C-reactive protein; ECOG, Eastern Cooperative Oncology Group performance status; HBeAg, hepatitis B e antigen; HBsAg, hepatitis B surface antigen; HBV, hepatitis B virus; HDV, hepatitis D virus; HCC, hepatocellular carcinoma; IFN, interferon; INR, international normalized ratio; LDH, lactate dehydrogenase; LTX, liver transplantation; MASLD, metabolic dysfunction–associated steatotic liver disease; MELD, Model for End-Stage Liver Disease; MetALD, metabolic dysfunction–associated steatotic liver disease with increased alcohol intake; NA, nucleos(t)ide analogue.

#### Characterization of HDV-HCC and HBV-HCC

Patients diagnosed with HDV-HCC presented with smaller tumors, less frequent vascular invasion, and more frequent fulfillment of the Milan criteria (all *p*<0.01; Table 1B). BCLC stage distribution did not differ significantly between groups (*p*=0.09). Histological tumor grade was comparable between groups, with most tumors being moderately differentiated (Table 1B).

**TABLE 1B:** Tumor characteristics of HDV-associated and HBV-associated hepatocellular carcinoma at the time of diagnosis

	HDV-HCC (n=54)	HBV-HCC (n=108)	*p*
Number of lesions (%, n)			
1	53.3% (24)	52.5% (53)	
2	26.7% (12)	23.8% (24)	
3	6.7% (3)	7.9% (8)	0.68
4	6.7% (3)	3.0% (3)	
5	0	0	
>6	6.7% (3)	12.9% (13)	
Largest lesion diameter (mm), mean±SD (range)	34.5±19.5 (80–95)	49.1±36.5 (5.5–170)	<0.01
Portal vein invasion (%, n)	2.3% (1)	19% (19)	<0.01
Suspicious lymph nodes (%, n)	27.3% (12)	29.6% (29)	0.78
Satellite lesions (%, n)	2.3% (1)	9.5% (9)	0.17
Extrahepatic lesions (%, n)	4.8% (2)	9.8% (8)	0.49
Within Milan criteria (%, n)	66.7% (34)	43.0% (46)	<0.01
BCLC stage (%, n)			
A	57.7% (30)	44.4% (48)	
B	28.8% (15)	33.3% (36)	0.09
C	5.8% (3)	18.5% (20)	
D	7.7% (4)	3.7% (4)	
Histological grad (%, n)			
G1	28.1% (24)	23.4% (15)	
G2	62.5% (20)	62.5% (40)	0.83
G3	9.4% (3)	12.5% (8)	
G4	0	1.6% (1)	

*Note:* Continuous variables are presented as mean±standard deviation (SD) with range. Categorical variables are presented as percentages (n). *p*-values <0.05 were considered statistically significant.

Abbreviations: BCLC, Barcelona Clinic Liver Cancer; HBV, hepatitis B virus; HCC, hepatocellular carcinoma; HDV, hepatitis D virus.

#### Virological status at the time of HCC diagnosis

At the time of HCC diagnosis, nucleos(t)ide analogue therapy was comparable between HBV-HCC and HDV-HCC patients (*p*=0.33). A subset of HBV-HCC patients (n=19) exhibited elevated HBV DNA levels (>20,000 IU/mL), primarily in untreated individuals. HBV DNA levels correlated with AST (*p*<0.01) but not with tumor diameter (*p*=0.77). In HDV-HCC, HDV RNA status was available for 41 patients; quantitative HDV RNA measurements (n=24) demonstrated wide interindividual variability (Table 1A). HDV RNA levels were not significantly associated with AST (*p*=0.45) or tumor diameter (*p*=0.42).

#### Analysis of the long-term follow-up

Following the diagnosis of HCC, patients were observed for a mean period of 4.1±5.1 years. Transarterial chemoembolization was the most common initial therapy in both groups (Supplemental Tables S2A, S2B, https://links.lww.com/HC9/C506). Liver transplantation occurred more often in HDV-HCC (Supplemental Table S3, https://links.lww.com/HC9/C506). Overall mortality did not differ between groups (*p*=0.39), with the majority of deaths being liver-related. Thirty-four patients underwent transplantation and were subsequently monitored for a mean period of 7.0±5.4 years; no post-transplant deaths occurred. Post-transplant tumor recurrence was observed exclusively in 2 patients with HBV-HCC (*p*=0.49). HBV-HCC patients underwent more tumor-directed treatment lines than HDV-HCC patients (Supplemental Tables S2, S3, https://links.lww.com/HC9/C506). However, treatment regimens were heterogeneous, with some patients receiving up to seven distinct therapeutic interventions. Kaplan–Meier analysis showed comparable survival between HBV-HCC and HDV-HCC (log-rank *p*=0.879; Figure 2).

**FIGURE 2:**
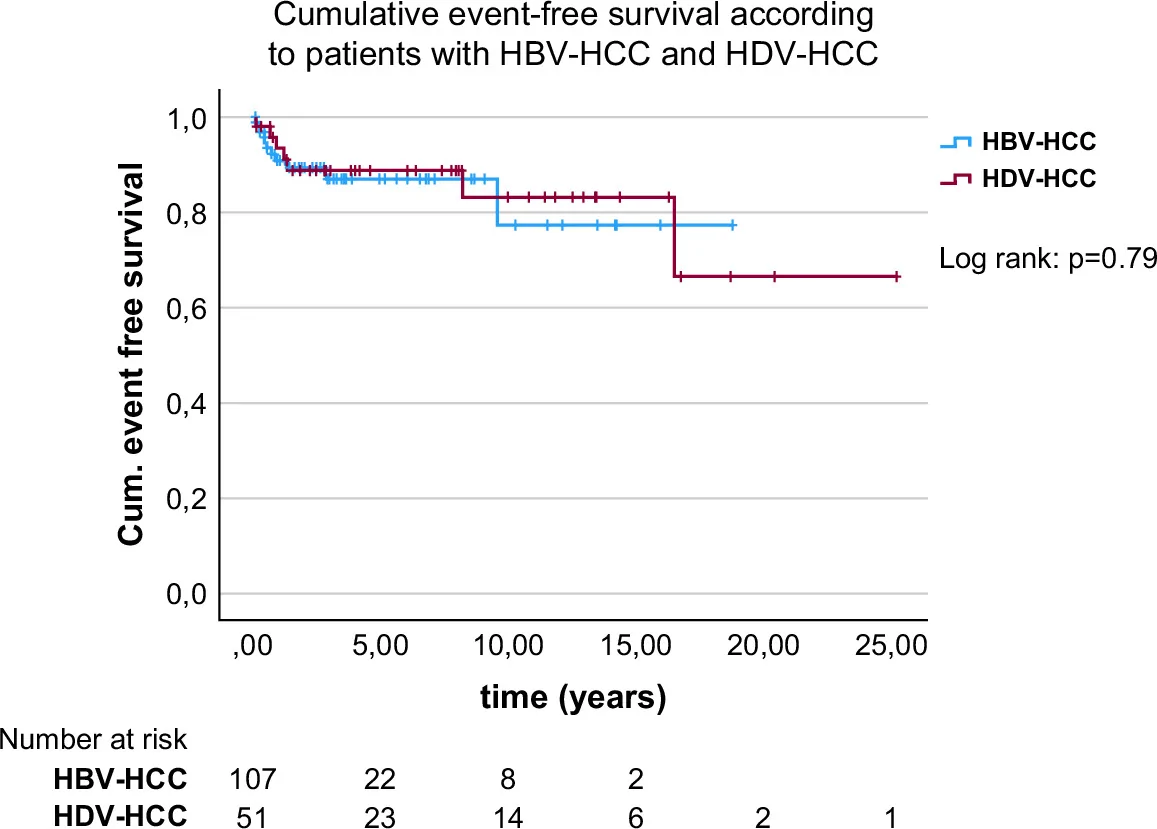
Cumulative event-free survival of patients with HBV-associated HCC and HDV-associated HCC, showing no significant difference between groups (*p*=0.79). Abbreviations: HBV, hepatitis B virus; HCC, hepatocellular carcinoma; HDV, hepatitis D virus.

Overall, HDV-HCC patients exhibited more advanced liver disease but smaller tumors and similar survival rates compared with HBV-HCC. We therefore next examined whether systemic inflammatory mediator (SIM) profiles differed in their association with clinical parameters between HDV-HCC and HBV-HCC.

### Distinct associations of SIM profiles with clinical parameters in HDV-HCC and HBV-HCC

Initially, we compared soluble inflammatory mediators with clinical parameters in a subgroup of patients with HDV-associated HCC (HDV-HCC; n=30) and HBV-associated HCC (HBV-HCC; n=50). Clinical differences between groups were preserved in this subgroup (Supplemental Table S4A, https://links.lww.com/HC9/C506). In HDV-HCC, no strong correlations between AST and SIMs were observed. In contrast, in HBV-HCC, AST strongly correlated (*r*>0.50) with multiple SIMs, including IL-6, IL-8, IL-10, MCP1, OPG, HGF, and TNFSF14 (Supplemental Figure S1, https://links.lww.com/HC9/C506). A stronger association with clinical parameters in HBV-HCC than in HDV-HCC was also evident in the correlation matrices (Supplemental Figures S2A, S2B, https://links.lww.com/HC9/C506). Overall, SIMs appeared to be more strongly associated with markers of liver injury and inflammation in HBV-HCC than in HDV-HCC. To further characterize etiology-associated systemic inflammatory signatures, comparative SIM profiling analyses were performed.

### Comparative SIM profiling in HDV-associated and HBV-associated HCC and control groups

To characterize the systemic inflammatory profiles associated with HCC in detail, a comparative analysis of the SIM profiles of HBV-HCC (n=50), HDV-HCC (n=30), and matched control patients (HBV=19, HDV=17) was performed. Compared with their respective non-HCC controls, HBV-HCC exhibited a broader inflammatory response, with 40 upregulated SIMs, whereas 23 SIMs were upregulated in HDV-HCC (Table 2). SCF was the only downregulated mediator in both comparisons. Twenty-one of the 23 SIMs upregulated in HDV-HCC versus HDV non-HCC controls were likewise upregulated in HBV-HCC versus HBV non-HCC controls, whereas CCL28 and CD6 were uniquely increased in HDV-HCC (Table 2).

**TABLE 2:** Differentially upregulated soluble immune mediators (SIMs) across HDV-associated HCC, HBV-associated HCC, and corresponding non-HCC control cohorts

HDV-HCC vs. HBV-HCC			HDV-HCC vs. HDV non-HCC			HBV-HCC vs. HBV non-HCC			HDV non-HCC vs. HBV non-HCC		
*p*<0.001	*p*<0.01	*p*<0.05	*p*<0.001	*p*<0.01	*p*<0.05	*p*<0.001	*p*<0.01	*p*<0.05	*p*<0.001	*p*<0.01	*p*<0.05
CD244	VEGFA	CXCL5	ARTN	CD244	CCL11	ARTN	CCL25	CCL19		CD244	CCL19
CD6	SLAMF1	CDCP1	FGF5	CCL28	CCL20	CCL11	CSF1	CD40			CD6
DNER	uPA	TNFb	IL17A	CD6	CCL25	CCL20	FGF21	CD5			CD8A
FGF21	EN-RAGE	IL20RA	PDL1	IL13	IL15RA	CD244	EBP1	CDCP1			IL10
SCF	IL2	FGF5	SLAMF1	LIFR	IL18R1	CD8A	IL6	IL10RA			LAPTGFbeta
	IL10	LIFR	IL10RB	TSLP	MMP10	FGF5	IL20RA	IL15RA			TNFb
	OSM		CD5	GDNF	IL20RA	GDNF	IL2RB	IL17C			uPA
	LIF		uPA	HGF		IL10RB	IL33	NT3			LIFR
			SCF			IL13	LAPTGFbeta	TGFalpha			ARTN
						IL17A	SCF	TNF			CCL11
						IL18R1	SLAMF1	TNFSF14			CCL25
						LIFR					CXCL6
						MMP10					PDL1
						PDL1					TNF
						TSLP					
						UPA					
						OPG					
						CXCL6					
						HGF					

*Note: p*-values were calculated using the Mann–Whitney *U* test. Significance levels are indicated as *p* < 0.05, *p* < 0.01, and *p* < 0.001.

Soluble immune mediator (SIM) levels differed significantly among the HDV-HCC, HBV-HCC, and their corresponding non-HCC control cohorts. Red indicates higher SIM levels in the first group compared with the second group. Blue indicates lower SIM levels in the first group compared with the second group.

Abbreviations: HBV, hepatitis B virus; HCC, hepatocellular carcinoma; HDV, hepatitis D virus; SIMs, soluble immune mediators.

To assess the robustness of the observed SIM differences, additional analyses, including false discovery rate (FDR) correction and adjustment for liver disease severity, were performed. Following Benjamini–Hochberg correction, the number of significantly altered mediators decreased across all comparisons. In the HDV-HCC versus HDV non-HCC comparison, 17 differentially expressed SIMs remained significant after FDR correction, comprising 16 upregulated mediators and the downregulated mediator SCF. Likewise, 33 differentially expressed SIMs remained significant in the HBV-HCC versus HBV non-HCC comparison, including 32 upregulated mediators and the downregulated mediator SCF (Tables 3A, 3B). In the direct comparison between HDV-HCC and HBV-HCC, 6 mediators remained significantly enriched in HDV-HCC (CD244, CD6, DNER, IL-10, SLAMF1, and uPA), whereas 5 mediators remained elevated in HBV-HCC after FDR correction (FGF21, SCF, LIF, VEGFA, and OSM) (Table 3C). Notably, several of these mediators, including CD244, CD6, uPA, and IL-10, were also differentially expressed in the exploratory comparison between HDV and HBV patients without HCC, although the latter analysis was not subjected to FDR correction because of the limited number of control samples (Table 2).

**TABLE 3A:** Differentially expressed soluble immune mediators between HDV non-HCC and HDV-HCC after FDR correction and MELD adjustment

SIM	Higher in	Raw *p*-value	FDR *q*-value	β coefficient (HCC status)	*p* after MELD adjustment	Interpretation
ARTN	HDV-HCC	0.0001	0.0012	0.558	0.0001	Preserved
CD5	HDV-HCC	0.0001	0.0012	0.421	0.003	Preserved
FGF5	HDV-HCC	0.0001	0.0012	0.481	0.001	Preserved
IL17A	HDV-HCC	0.0001	0.0012	0.498	0.0001	Preserved
PDL1	HDV-HCC	0.0001	0.0012	0.416	0.002	Preserved
SCF	HDV-non-HCC	0.0001	0.0012	−0.454	0.001	Preserved
SLAMF1	HDV-HCC	0.0001	0.0012	0.398	0.003	Preserved
uPA	HDV-HCC	0.0001	0.0012	0.367	0.005	Preserved
IL10RB	HDV-HCC	0.0001	0.0012	0.463	0.0001	Preserved
CCL28	HDV-HCC	0.001	0.0077	0.471	0.0001	Preserved
CD6	HDV-HCC	0.001	0.0077	0.370	0.006	Preserved
HGF	HDV-HCC	0.001	0.0077	0.441	0.002	Preserved
LIFR	HDV-HCC	0.002	0.0142	0.390	0.008	Preserved
CD244	HDV-HCC	0.003	0.0184	0.316	0.017	Preserved
IL13	HDV-HCC	0.003	0.0184	0.425	0.005	Preserved
TSLP	HDV-HCC	0.005	0.0288	0.394	0.009	Preserved
GDNF	HDV-HCC	0.006	0.0325	0.331	0.025	Preserved
IL18R1	HDV-HCC	0.011	0.0562	—	—	Attenuated after FDR
MMP10	HDV-HCC	0.012	0.0581	—	—	Attenuated after FDR
CCL11	HDV-HCC	0.013	0.0598	—	—	Attenuated after FDR
CCL20	HDV-HCC	0.015	0.0627	—	—	Attenuated after FDR
CCL25	HDV-HCC	0.015	0.0627	—	—	Attenuated after FDR
IL15RA	HDV-HCC	0.016	0.0640	—	—	Attenuated after FDR
IL20RA	HDV-HCC	0.018	0.0690	—	—	Attenuated after FDR

*Note:* *p*-values were corrected for multiple testing using the Benjamini–Hochberg false discovery rate method. Multivariable linear regression analyses were adjusted for MELD score. HCC status was coded as non-HCC=0 and HCC=1; positive β coefficients indicate higher soluble immune mediator levels in HDV-HCC, whereas negative β coefficients indicate higher levels in HDV non-HCC.

Abbreviations: FDR, false discovery rate; HBV, hepatitis B virus; HCC, hepatocellular carcinoma; HDV, hepatitis D virus; MELD, Model for End-Stage Liver Disease; SIM, soluble immune mediator.

**TABLE 3B:** Differentially expressed soluble immune mediators between HBV non-HCC and HBV-HCC after FDR correction and MELD adjustment

SIM	Higher in	Raw *p*-value	FDR *q*-value	β coefficient (HCC status)	*p* after MELD adjustment	Interpretation
ARTN	HBV-HCC	0.0001	0.0005	0.395	0.0001	Preserved
CCL11	HBV-HCC	0.0001	0.0005	0.302	0.006	Preserved
CCL20	HBV-HCC	0.0001	0.0005	0.409	0.0001	Preserved
CD244	HBV-HCC	0.0001	0.0005	0.277	0.013	Preserved
CD8A	HBV-HCC	0.0001	0.0005	0.406	0.0001	Preserved
CXCL6	HBV-HCC	0.0001	0.0005	0.358	0.003	Preserved
FGF5	HBV-HCC	0.0001	0.0005	0.689	0.0001	Preserved
GDNF	HBV-HCC	0.0001	0.0005	0.341	0.004	Preserved
HGF	HBV-HCC	0.0001	0.0005	0.318	0.008	Preserved
IL10RB	HBV-HCC	0.0001	0.0005	0.360	0.001	Preserved
IL13	HBV-HCC	0.0001	0.0005	0.460	0.0001	Preserved
IL17A	HBV-HCC	0.0001	0.0005	0.509	0.0001	Preserved
IL18R1	HBV-HCC	0.0001	0.0005	0.380	0.001	Preserved
LIFR	HBV-HCC	0.0001	0.0005	0.488	0.0001	Preserved
MMP10	HBV-HCC	0.0001	0.0005	0.342	0.002	Preserved
OPG	HBV-HCC	0.0001	0.0005	0.411	0.0001	Preserved
PDL1	HBV-HCC	0.0001	0.0005	0.341	0.001	Preserved
TSLP	HBV-HCC	0.0001	0.0005	0.413	0.0001	Preserved
uPA	HBV-HCC	0.0001	0.0005	0.415	0.0001	Preserved
IL2RB	HBV-HCC	0.001	0.0042	0.350	0.005	Preserved
IL33	HBV-HCC	0.001	0.0042	0.341	0.006	Preserved
LAPTGFbeta1	HBV-HCC	0.001	0.0042	0.334	0.005	Preserved
CCL25	HBV-HCC	0.002	0.0080	0.309	0.008	Preserved
SLAMF1	HBV-HCC	0.003	0.0110	0.241	0.029	Preserved
CSF1	HBV-HCC	0.004	0.0131	0.264	0.030	Preserved
EBP1	HBV-HCC	0.004	0.0131	0.291	0.019	Preserved
IL20RA	HBV-HCC	0.004	0.0131	0.243	0.040	Preserved
SCF	HBV-non-HCC	0.005	0.0159	−0.240	0.050	Preserved
FGF21	HBV-HCC	0.007	0.0215	0.304	0.016	Preserved
NT3	HBV-HCC	0.010	0.0288	0.326	0.009	Preserved
CDCP1	HBV-HCC	0.012	0.0335	0.250	0.045	Preserved
IL6	HBV-HCC	0.009	0.0267	0.241	0.052	Trend after MELD adjustment
IL15RA	HBV-HCC	0.014	0.0379	0.154	0.170	Attenuated after MELD adjustment
TNF	HBV-HCC	0.020	0.0526	—	—	Attenuated after FDR
IL10RA	HBV-HCC	0.022	0.0529	—	—	Attenuated after FDR
TGFalpha	HBV-HCC	0.022	0.0529	—	—	Attenuated after FDR
CD5	HBV-HCC	0.023	0.0529	—	—	Attenuated after FDR
IL17C	HBV-HCC	0.023	0.0529	—	—	Attenuated after FDR
TNFSF14	HBV-HCC	0.023	0.0529	—	—	Attenuated after FDR
CD40	HBV-HCC	0.024	0.0539	—	—	Attenuated after FDR
CCL19	HBV-HCC	0.025	0.0548	—	—	Attenuated after FDR

*Note:* *p*-values were corrected for multiple testing using the Benjamini–Hochberg false discovery rate method. Multivariable linear regression analyses were adjusted for MELD score. HCC status was coded as non-HCC=0 and HCC=1; positive β coefficients indicate higher soluble immune mediator levels in HBV-HCC, whereas negative β coefficients indicate higher levels in HBV non-HCC.

Abbreviations: FDR, false discovery rate; HBV, hepatitis B virus; HCC, hepatocellular carcinoma; MELD, Model for End-Stage Liver Disease; SIM, soluble immune mediator.

**TABLE 3C:** Differentially expressed soluble immune mediators between HDV-HCC and HBV-HCC after FDR correction and MELD adjustment

SIM	Higher in	Raw *p*-value	FDR *q*-value	β coefficient (etiology)	*p* after MELD adjustment	Interpretation
CD244	HDV-HCC	0.0001	0.0015	−0.305	0.002	Preserved
CD6	HDV-HCC	0.0001	0.0015	−0.328	0.002	Preserved
DNER	HDV-HCC	0.0001	0.0015	−0.343	0.001	Preserved
FGF21	HBV-HCC	0.0001	0.0015	0.300	0.008	Preserved
SCF	HBV-HCC	0.0001	0.0015	0.330	0.003	Preserved
LIF	HBV-HCC	0.001	0.0015	0.284	0.013	Preserved
IL10	HDV-HCC	0.002	0.0184	−0.256	0.016	Preserved
SLAMF1	HDV-HCC	0.002	0.0184	−0.227	0.021	Preserved
VEGFA	HBV-HCC	0.002	0.0184	0.285	0.013	Preserved
OSM	HBV-HCC	0.002	0.0184	0.267	0.020	Preserved
uPA	HDV-HCC	0.004	0.0335	−0.225	0.027	Preserved
IL2	HBV-HCC	0.007	0.0537	0.232	0.037	Attenuated after FDR
EN-RAGE	HBV-HCC	0.009	0.0637	0.212	0.060	Trend after MELD adjustment
FGF5	HBV-HCC	0.011	0.0723	0.256	0.025	Attenuated after FDR
IL20RA	HBV-HCC	0.012	0.0736	0.220	0.056	Attenuated
CXCL5	HBV-HCC	0.014	0.0805	0.220	0.049	Attenuated after FDR
TNFβ	HDV-HCC	0.018	0.0974	−0.178	0.108	Attenuated
CDCP1	HDV-HCC	0.021	0.1073	−0.185	0.108	Attenuated
LIFR	HDV-HCC	0.024	0.1139	−0.175	0.091	Attenuated

*Note:* *p*-values were corrected for multiple testing using the Benjamini–Hochberg false discovery rate method. Multivariable linear regression analyses were adjusted for MELD score. Etiology was coded as HDV-HCC=1 and HBV-HCC=2; negative β coefficients indicate higher SIM levels in HDV-HCC and positive β coefficients indicate higher levels in HBV-HCC.

Abbreviations: FDR, false discovery rate; HBV, hepatitis B virus; HCC, hepatocellular carcinoma; HDV, hepatitis D virus; MELD, Model for End-Stage Liver Disease; SIM, soluble immune mediator.

Multivariable linear regression analyses adjusted for MELD score were subsequently performed for FDR-significant mediators to address potential confounding by liver disease severity. Importantly, all FDR-significant mediators distinguishing HDV-HCC from HDV non-HCC remained significantly associated with HCC status after MELD adjustment. Similarly, most FDR-significant mediators differentiating HBV-HCC from HBV non-HCC remained significantly associated with HCC status, whereas IL15RA lost statistical significance and IL6 showed only a statistical trend (*p*=0.052), suggesting a partial influence of liver disease severity. In the direct comparison between HDV-HCC and HBV-HCC, the principal FDR-significant inflammatory differences likewise remained significantly associated with disease group after MELD adjustment. Notably, IL2, FGF5, and CXCL5, which did not remain significant after FDR correction, remained statistically significant after MELD adjustment (Table 3C).

Given the substantial influence of advanced cirrhosis on systemic inflammatory signaling, an additional sensitivity analysis restricted to cirrhotic HCC patients was performed (HDV-HCC n=28; HBV-HCC n=38). Importantly, all principal inflammatory differences identified in the primary comparison between HDV-HCC and HBV-HCC were confirmed in the cirrhosis-restricted analysis (Supplemental Table S5, https://links.lww.com/HC9/C506). In addition, several further SIMs demonstrated concordant differential expression, whereas exploratory findings included CXCL10, CXCL11, and TRAIL in HDV-HCC and IL2RB and BetaNGF in HBV-HCC. Because only a limited number of etiology-matched non-HCC controls had documented cirrhosis (HDV non-HCC: n=7; HBV non-HCC: n=5), cirrhosis-restricted analyses were limited to the HCC cohorts to avoid substantially underpowered comparisons. Collectively, these findings indicate robust etiology-associated systemic inflammatory signatures in HBV-HCC and HDV-HCC and provided the basis for subsequent pathway and protein–protein interaction analyses to further characterize their potential biological relevance.

### Integrated pathway and protein–protein interaction analyses

We next extracted significantly differentially expressed SIMs after FDR correction and MELD adjustment and performed enrichment analyses using g:Profiler and STRING to explore associated signaling pathways and protein–protein interactions. Out of the 30 SIMs upregulated in HBV-HCC versus HBV non-HCC, 13 overlapped with the HDV-HCC versus HDV non-HCC comparison (Figure 3A). STRING analyses identified an IL-13, IL-17A–associated network involving positive regulation of IL-10 production (Biological Process GO), regulation of IL-6 production, signaling by IL-4 and IL-13 (REACTOME), cytokine–cytokine receptor interaction pathways (KEGG), signaling by interleukins, including JAK–STAT signaling pathways and Interleukin-1 family (Figure 4). These shared signaling patterns may indicate common inflammatory features associated with the transition from chronic viral hepatitis to HCC. Seventeen SIMs were preferentially upregulated in HBV-HCC compared with HBV non-HCC (Figure 3A) and were mainly associated with an IL-33, CD8a, CCL20, and CCL11-centered network, connected to chemokine-mediated signaling pathway and adaptive immunity (STRING). These networks were dominated by pro-inflammatory signaling pathways, suggesting a comparatively stronger cytokine-associated systemic inflammatory profile in HBV-HCC. This included viral protein interaction with cytokine and cytokine receptor, as well as TNF-signaling (KEGG), but also myeloid leukocyte migration and neutrophil chemostaxis, indicating also association with innate immunity (Figure 4).

**FIGURE 3:**
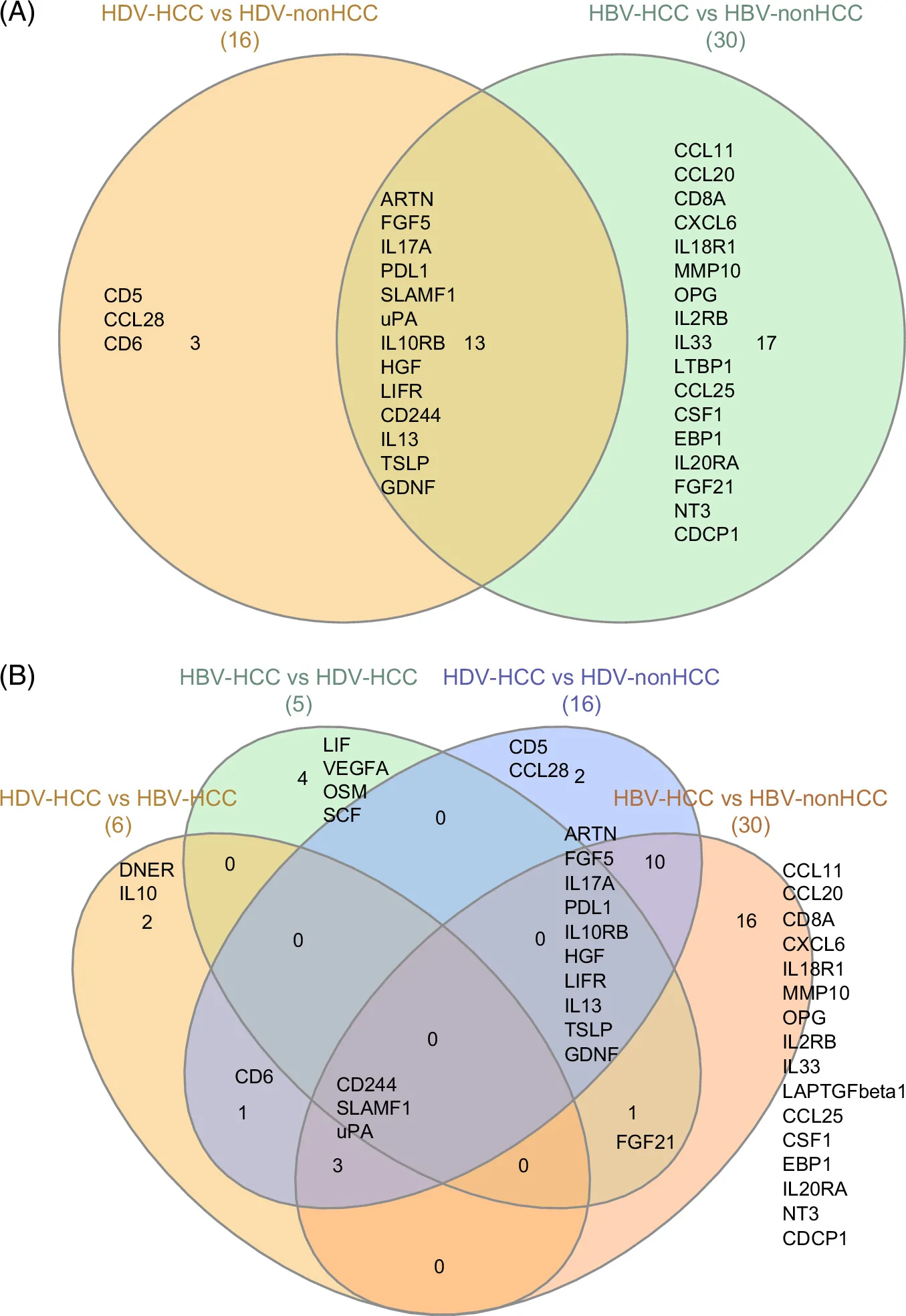
(A) Venn diagram illustrating upregulated SIMs in the intra-etiology HCC versus non-HCC comparisons and their overlap. (B) Venn diagram illustrating upregulated SIMs across study subgroups and their overlaps. Abbreviations: HCC, hepatocellular carcinoma; HDV, hepatitis D virus; SIMs, soluble inflammatory mediators.

FIGURE 4(A) Network interaction analysis of persistently altered SIMs shared between HBV-associated HCC versus HBV non-HCC and HDV-associated HCC versus HDV non-HCC. Network was created via STRING, Biological Process GO, KEGG, and REACTOME. (B) Network interaction analysis of persistently altered SIMs exclusively upregulated in HBV-associated HCC compared with HBV non-HCC. Network was created via STRING, Biological Process GO, KEGG, and REACTOME. (C) Network interaction analysis of persistently altered SIMs upregulated in HDV-associated HCC compared with HBV-associated HCC. Network was created via STRING and Biological Process GO. (D) Network interaction analysis of persistently altered SIMs upregulated in HBV-associated HCC compared with HDV-associated HCC. Network was created via STRING, Biological Process GO, KEGG, and REACTOME. Abbreviations: GO, Gene Ontology; HBV, hepatitis B virus; HCC, hepatocellular carcinoma; HDV, hepatitis D virus; KEGG, Kyoto Encyclopedia of Genes and Genomes; SIMs, soluble immune mediators.

**Figure:**
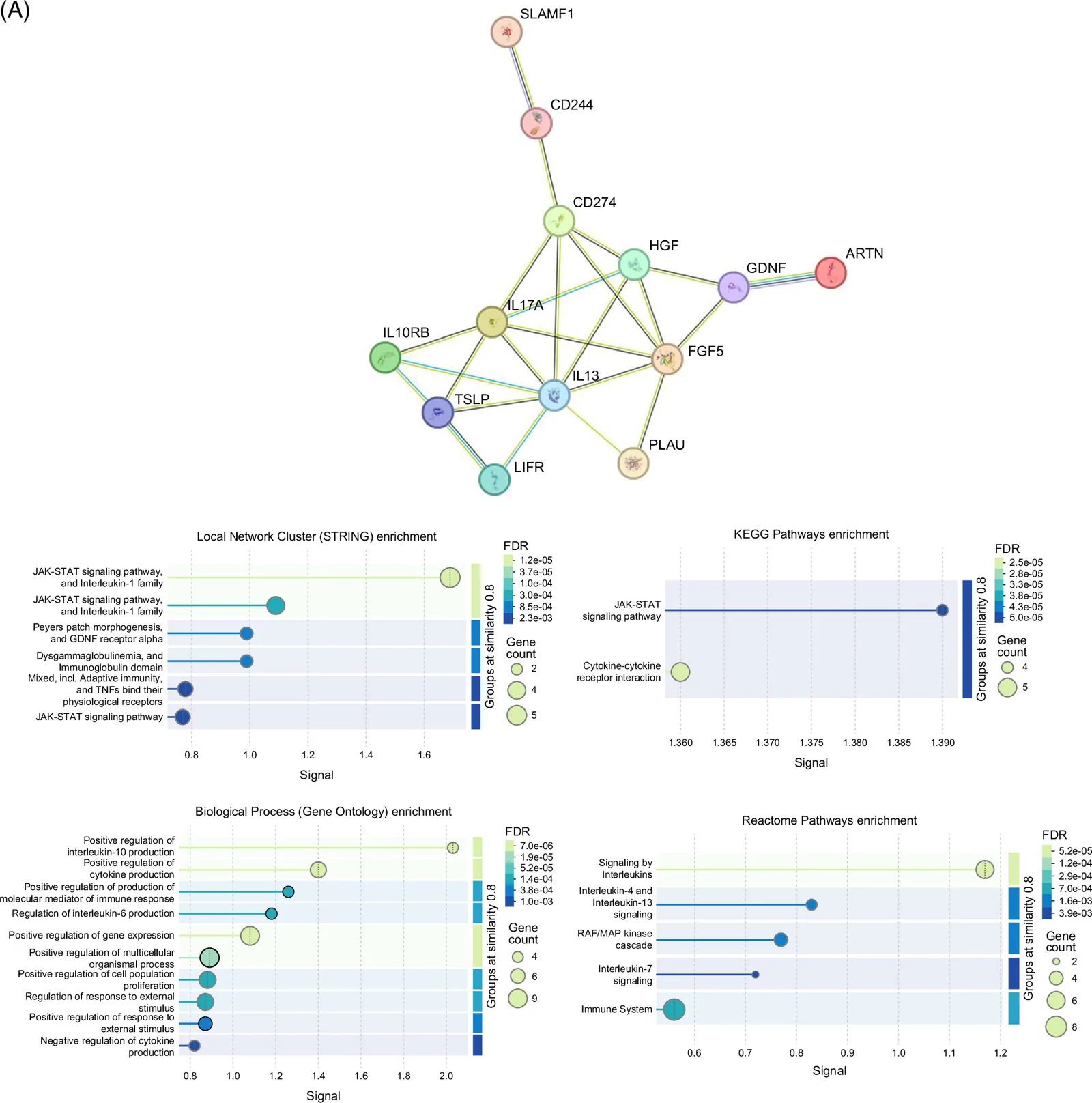

**Figure:**
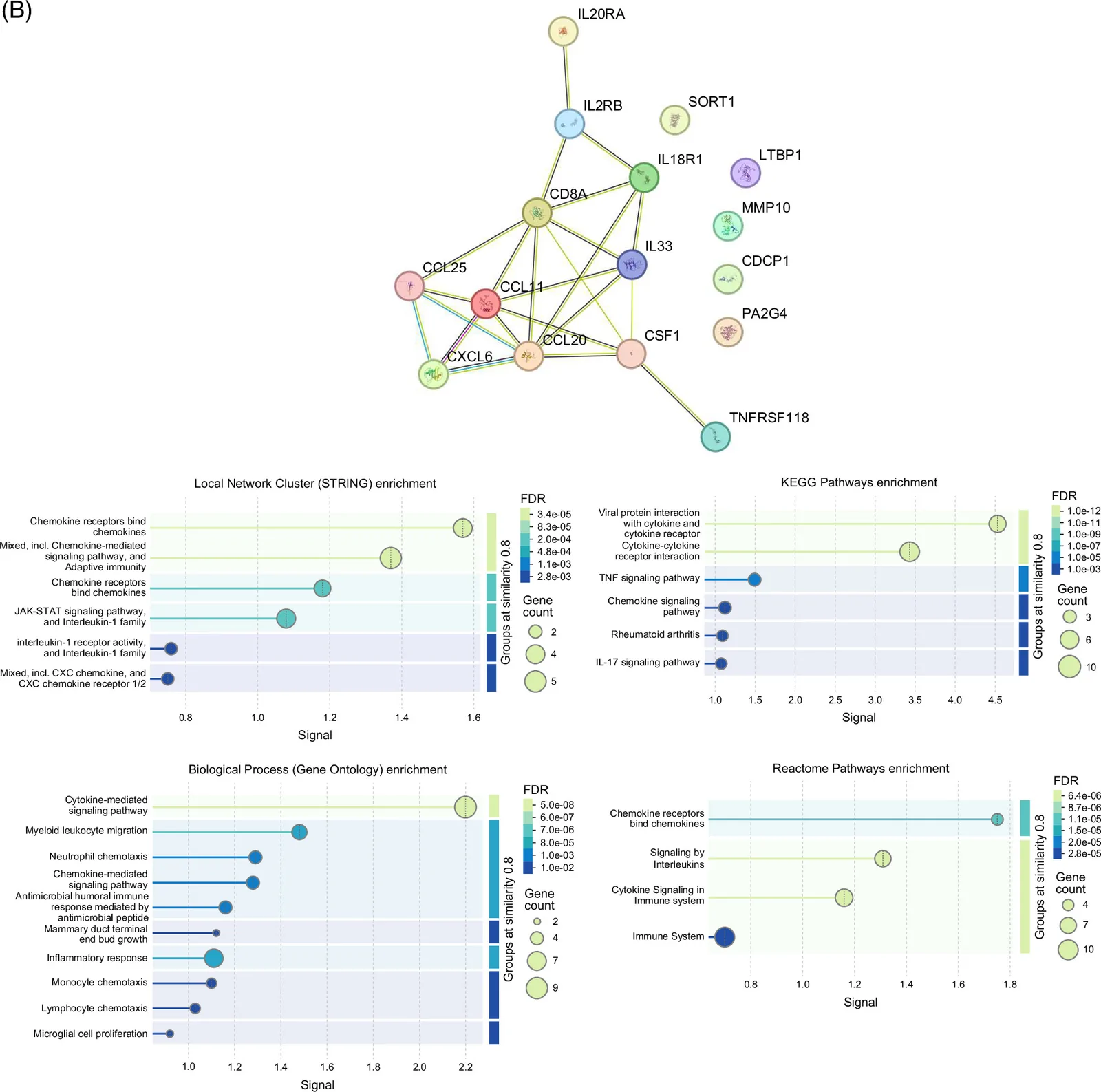

**Figure:**
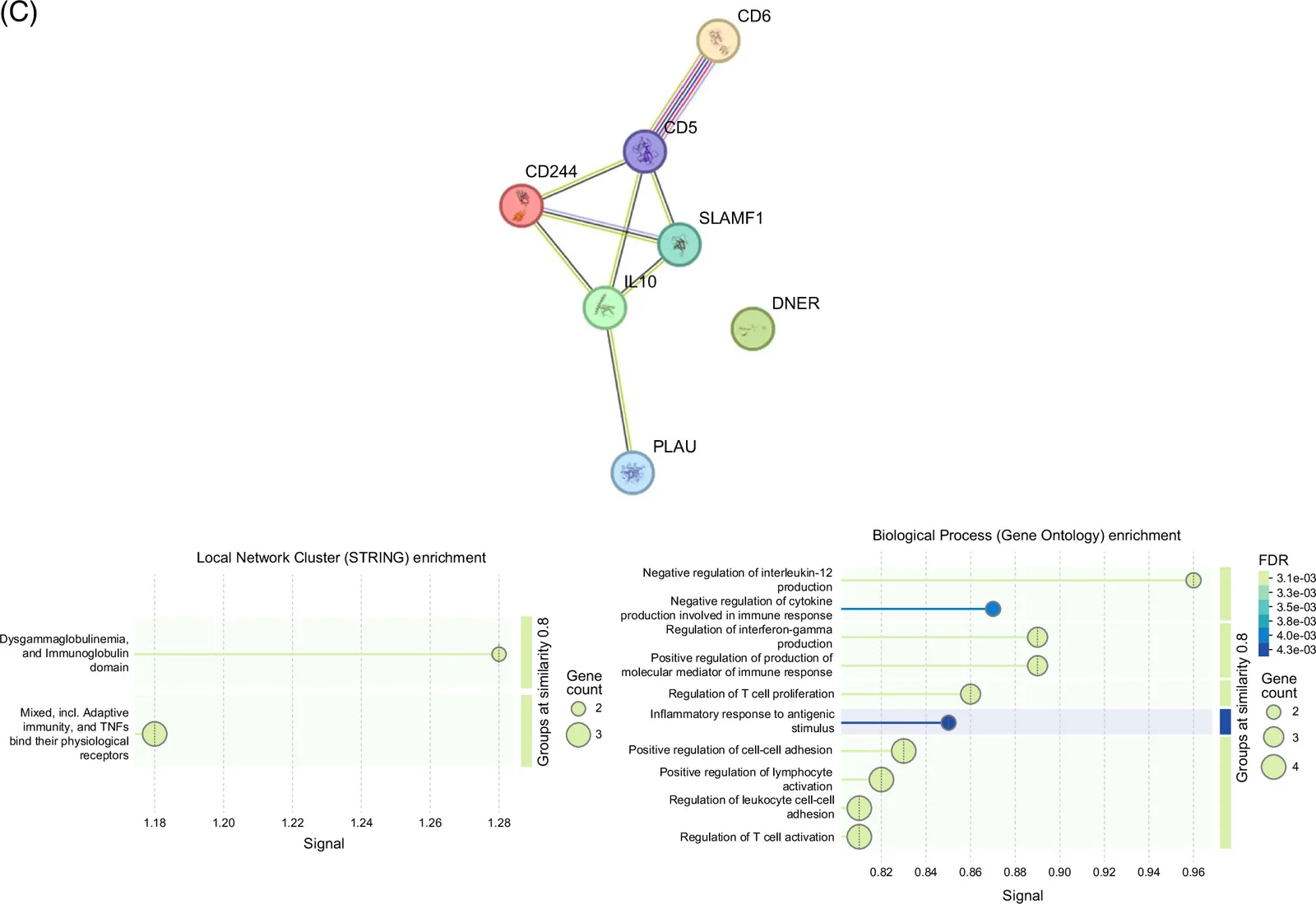

**Figure:**
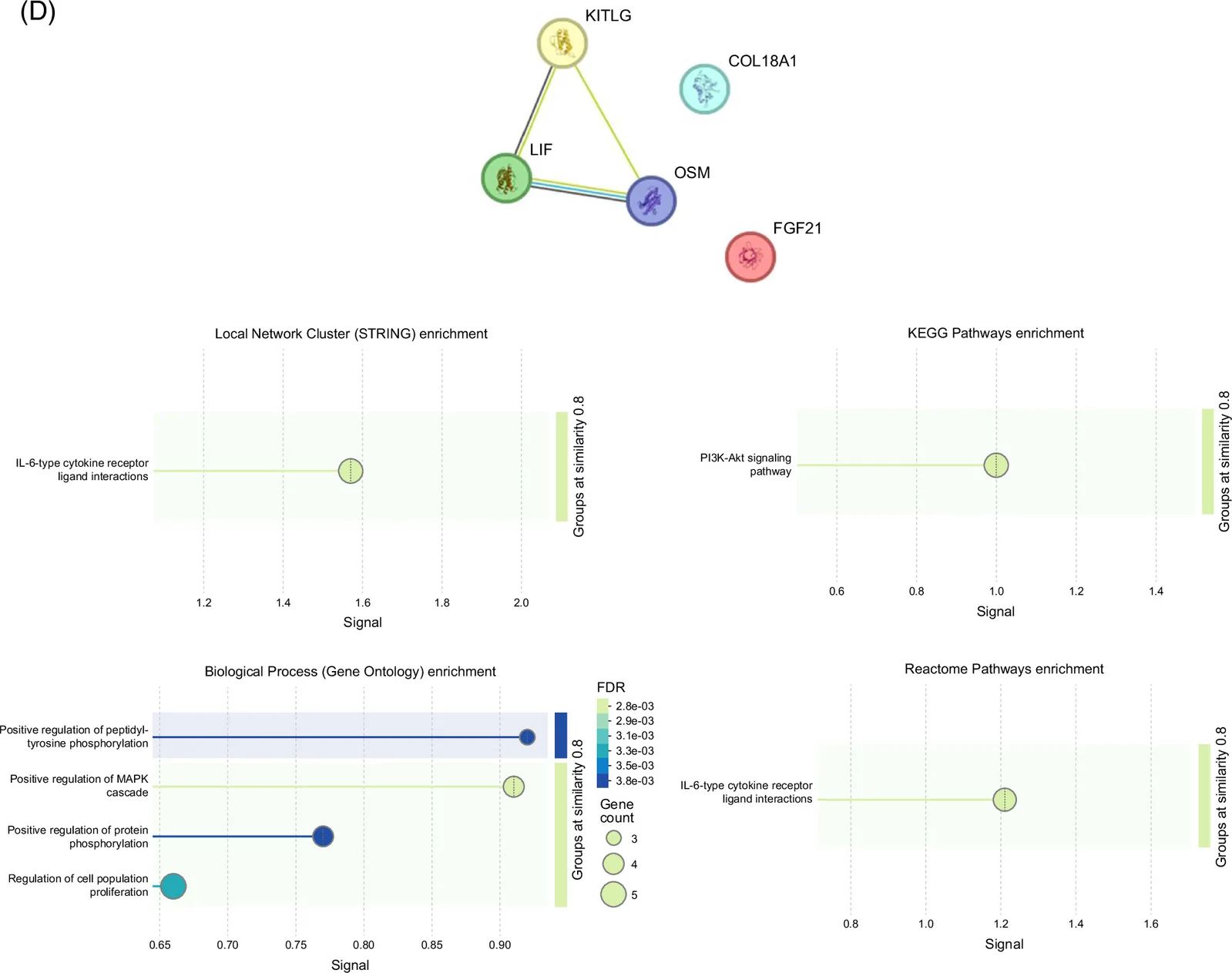

Direct comparison of HDV-HCC and HBV-HCC revealed upregulation of 8 SIMs, including the uniquely enriched mediators CCL28 and CD5 (Figure 3B). These SIMs were enriched for pathways related to the regulation of interferon-γ and cytokine production, as well as the negative regulation of IL-12. STRING analysis suggested an IL-10–associated immunoregulatory network involving SLAMF1, CD244, PLAU (uPA), CD5, and CD6, together with regulation of T-cell proliferation. No significant pathway enrichment was detected in REACTOME and KEGG (Figure 4). These findings suggest a comparatively stronger IL-10–associated immunoregulatory signaling profile in HDV-HCC.

Five SIMs were upregulated in HBV-HCC (Figure 3B). Pathway enrichment analyses indicated Pi3K–Akt signaling and phosphorylation signaling, associated with cell growth and proliferation activity. STRING and REACTOME network analyses showed an IL-6-type cytokine receptor ligand interaction (Figure 4). Together, these findings suggest a comparatively stronger IL-6-associated inflammatory signaling profile in HBV-HCC.

Taken together, these findings indicate partly overlapping systemic inflammatory pathway alterations associated with the transition from non-HCC to HCC in both HBV and HDV. In addition, pathway enrichment analyses revealed a broader IL-6–associated inflammatory signaling profile in HBV-HCC, whereas HDV-HCC was characterized by a comparatively stronger IL-10–associated immunoregulatory profile with reduced IL-12 signaling.

## DISCUSSION

Hepatocellular carcinoma (HCC) represents a major health burden and is one of the most common causes of cancer-related deaths worldwide.^6^ In this study, we compared clinical characteristics and systemic inflammatory profiles in patients with HBV-associated and HDV-associated HCC. Hepatitis delta represents the most severe form of viral hepatitis with high progression rates to cirrhosis and consecutive decompensations.^3^,^9^ This is consistent with our findings, in which patients with CHD exhibited impaired liver function, a higher incidence of cirrhosis, and more previous episodes of hepatic decompensation than patients with CHB alone. Despite these unfavorable baseline characteristics, mortality rates after HCC diagnosis did not differ between HBV-HCC and HDV-HCC. Moreover, patients with HDV-HCC presented with smaller tumors and more frequently fulfilled Milan criteria. These findings are in line with previous studies, demonstrating that HDV-HCC is associated with more advanced liver disease but comparable tumor stage and survival outcomes compared with HBV-HCC.^10^,^11^,^12^ This may reflect earlier surveillance in HDV or intrinsic differences in the systemic inflammatory landscape between HBV-associated and HDV-associated HCC. In addition, no patients died following LTX, although liver function was more impaired in HDV-HCC patients, which underscores the clinical relevance of timely liver transplantation in this population.^19^ While previous studies have primarily focused on clinical characteristics, the underlying immunological differences between HBV-associated and HDV-associated HCC remain poorly understood. Here, we provide a comprehensive analysis of systemic inflammatory mediators (SIMs), offering novel insights into virus-specific systemic immune profiles associated with HCC. In contrast to previous reports,^11^ we observed significantly elevated levels of leukocytes, CRP, and AST in patients with HBV-HCC compared with HDV-HCC, suggesting that progression to HCC in HBV may be associated with enhanced systemic immune activation compared with HDV. Although HBV-HCC showed a greater number of upregulated SIMs than HDV-HCC, pathway analyses revealed overlapping inflammatory signaling pathways. These findings suggest that shared inflammatory pathways, including IL-13 and members of the IL-1 family, JAK–STAT signaling, as well as IL-6–related and IL-10–related pathways, may be associated with hepatocarcinogenesis across viral etiologies. Several SIMs overexpressed in both HBV-HCC and HDV-HCC were also reported in HCV-associated HCC, supporting the concept of common inflammatory mechanisms in virus-associated hepatocarcinogenesis.^20^ However, direct comparison of HBV-HCC and HDV-HCC demonstrated that these shared inflammatory pathways differed in their relative predominance. While HDV-HCC was characterized by a stronger IL-10–associated immunoregulatory signature, including an IL-10–centered interaction network, HBV-HCC preferentially exhibited enrichment of IL-6–associated, phosphorylation-related, and proliferation-related pathways, consistent with previous studies implicating IL-6 in inflammation-associated hepatocarcinogenesis.^21^ This was further supported by higher CRP and AST levels in HBV-HCC. These immunological differences align with recent tissue-based studies reporting distinct molecular signatures in HDV-associated versus HBV-associated HCC. While HDV-associated HCC has been linked to pathways involved in DNA repair, cell cycle regulation, and genomic instability, HBV-associated HCC appears to be more closely associated with metabolic processes and cell remodeling, further supporting the concept that HBV-related and HDV-related HCC represent biologically distinct entities.^22^ To determine whether these differences reflected etiology-associated immune alterations rather than differences in liver disease severity, we next examined the individual SIM profiles in greater detail. Importantly, these principal differences remained largely preserved after correction for multiple testing, MELD adjustment, and sensitivity analyses restricted to cirrhotic HCC patients, supporting that the observed inflammatory signatures are not solely explained by differences in liver disease severity.

HDV-HCC was characterized by preferential elevation of CD6, SLAMF1, DNER, CD244, and uPA, whereas CCL28 and CD5 were exclusively upregulated in HDV-HCC compared with HDV non-HCC controls. Interestingly, several mediators contributing to this HDV-associated immunoregulatory profile were already elevated in HDV non-HCC controls compared with HBV non-HCC controls. This overlap was most evident for the FDR-confirmed mediators CD244, IL-10, and uPA, whereas similar trends were observed for TNFβ and LIFR in the exploratory control comparison. While the exploratory comparison of HDV and HBV non-HCC controls was not subjected to FDR correction because of the limited number of control samples, it suggests that part of the inflammatory profile observed in HDV-HCC may reflect immune alterations already present during chronic HDV infection. Importantly, several of these mediators, particularly the FDR-confirmed markers CD244, CD6, and uPA, were further increased in HDV-HCC compared with HDV non-HCC controls, suggesting that the immune alterations already present during chronic HDV infection are further amplified during hepatocarcinogenesis. Together, these findings suggest that the comparatively stronger immunoregulatory systemic inflammatory profile observed in HDV-HCC may reflect both chronic HDV-associated immune alterations and HCC-associated inflammatory remodeling rather than representing exclusively HCC-associated inflammatory changes.

This interpretation is supported by previous studies demonstrating that several of the preferentially elevated SIMs identified in HDV-HCC have been linked to immune regulation, tumor progression, and cell–cell signaling in HCC and other malignancies.^23^,^24^,^25^,^26^,^27^,^28^,^29^ Likewise, increased CD244 and IL-10 have been associated with T-cell dysfunction and altered antiviral immune regulation in chronic HBV and HCV infection.^30^,^31^,^32^,^33^ Consistent with previous studies, TNF-β, uPA, and LIFR have been linked to fibrogenesis, chronic inflammation, and impaired hepatic regeneration.^34^,^35^,^36^ Our findings are in line with these observations, although TNF-β and LIFR should be interpreted cautiously, as they were not confirmed after FDR correction. Although comparable immunological data for HDV-associated HCC are largely unavailable, studies in chronic HDV infection provide important biological context for interpreting the systemic inflammatory profile observed in HDV-associated HCC. These studies have demonstrated increased cirrhosis rates and systemic inflammation, accompanied by enhanced innate immune activation but weak or absent antigen-specific CD4^+^ T-cell responses.^15^,^37^,^38^,^39^ Furthermore, CD8^+^ T cells in CHD are activated but functionally impaired, while reduced numbers and impaired function of MAIT cells further support T-cell dysfunction in this setting.^40^,^41^ Collectively, these findings suggest that chronic systemic immune activation combined with impaired adaptive immunity may contribute to the altered systemic immune regulation observed in chronic HDV infection. In addition, impaired adaptive immune responses in chronic HDV infection have been described as IL-10–dominant cytokine profiles.^42^ IL-10 is a potent immunosuppressive cytokine that inhibits IL-12 production, a key mechanism implicated in altered immune regulation in chronic inflammatory states.^43^ However, the contribution of IL-10–associated immune regulation to the systemic inflammatory profile observed in HDV-associated HCC remains insufficiently characterized. In our HDV-HCC cohort, interaction network and pathway analyses identified a comparatively stronger IL-10–associated systemic immunoregulatory profile, accompanied by enrichment of pathways involved in negative regulation of IL-12 production—a key cytokine for NK-cell activation—as well as regulation of T-cell proliferation and interferon-gamma signaling. These pathway findings are further supported by reports demonstrating reduced NK-cell numbers and impaired TNF-α and IFN-γ production in advanced HCC, consistent with compromised effector function.^44^

Taken together, these findings support the presence of 2 distinct systemic inflammatory signatures in HDV-HCC and HBV-HCC: an immunoregulatory profile in HDV-HCC and a pro-inflammatory profile in HBV-HCC. While the comparatively stronger pro-inflammatory systemic signature in HBV-HCC was accompanied by higher AST, CRP, and leukocyte levels, HDV-HCC exhibited a more immunoregulatory profile, suggesting that the systemic immune profile observed in HDV-associated HCC reflects both chronic HDV-associated immune dysregulation and HCC-associated inflammatory remodeling. These differences may have important clinical implications. Despite this immunoregulatory milieu, HDV-HCC patients in our cohort presented with significantly smaller tumors. This observation may reflect differences in surveillance or the presence of advanced cirrhosis, which has been associated with restricted tumor growth.^45^,^46^ Alternatively, the distinct systemic inflammatory profile in HDV-HCC may be associated with differences in tumor behavior compared with the pro-inflammatory, IL-6–associated profile observed in HBV-HCC, with potential implications for therapeutic responsiveness, particularly in the context of immune-based therapies.^44^,^45^,^46^ However, because SIM profiling was performed before tumor-directed treatment and no longitudinal treatment-response data were available, any association between these immune signatures and treatment response remains speculative and requires prospective evaluation. Previous studies have demonstrated that the efficacy of immunotherapies may vary depending on the underlying liver disease and tumor immune microenvironment.^47^ In this context, our findings raise the possibility that the immunoregulatory profile observed in HDV-HCC may have implications for responsiveness to immune-based therapies, although direct clinical evidence supporting this hypothesis is currently lacking. Notably, the observed IL-10–dominant and IL-12–suppressed signaling profile is in line with previous studies in chronic HDV infection, demonstrating impaired virus-specific T-cell function that was only partly restored by PD-1 blockade but more consistently enhanced by IL-12 supplementation.^48^ While our plasma-based SIM data do not permit conclusions regarding therapeutic responsiveness, the observed enrichment of pathways related to negative regulation of IL-12 production provides a rationale for further investigating IL-12–associated immunoregulatory pathways in HDV-associated HCC.

This study has several strengths and limitations. A major strength is the relatively large cohort of HBV-HCC and HDV-HCC patients with follow-up data. In addition, unbiased profiling of a broad panel of SIMs was performed in patient subgroups and matched controls. However, several limitations should be acknowledged. First, the retrospective design, heterogeneous viral load dynamics, and the use of different assays for HDV RNA quantification limit direct comparability across patients.^49^ Furthermore, HBV and HDV genotypes were not analyzed. Second, SIMs were measured in plasma and therefore reflect systemic inflammatory signatures rather than the local tumor microenvironment. Consequently, our findings should be interpreted as systemic immune states associated with HCC rather than direct measures of local tumor immunobiology. Because of the retrospective study design and limited availability of explant material, tissue-based validation by immunohistochemical or transcriptomic analyses could not be performed. Future prospective studies integrating plasma profiling with tissue-based approaches, including immunohistochemistry, spatial immune profiling, and transcriptomic analyses, are warranted. Such studies will be essential to determine whether the etiology-associated systemic immune signatures identified in our study are recapitulated within the hepatic tumor microenvironment. In addition, the direct comparison between HDV and HBV non-HCC controls should be interpreted with caution, as the limited number of control samples precluded FDR correction. Consequently, these analyses were considered exploratory and primarily served to provide context for the inflammatory differences observed between HDV-HCC and HBV-HCC.

Third, SIM profiling was performed at a single baseline time point before tumor-directed therapy, precluding assessment of longitudinal immune dynamics before HCC occurrence or during treatment. Owing to the retrospective design, serial plasma samples were only available for a limited number of patients, and heterogeneous treatment strategies resulted in subgroup sizes too small for meaningful treatment-specific analyses, including immune checkpoint inhibition responsiveness. Therefore, any potential relevance of distinct immune signatures for immunotherapy response remains speculative and requires prospective validation.

Finally, advanced cirrhosis itself profoundly alters systemic inflammatory pathways and therefore represents a potential confounder in the interpretation of circulating SIMs. To address this concern, we performed multivariable analyses adjusted for MELD score and additional sensitivity analyses restricted to cirrhotic HCC patients in the direct comparison between HDV-HCC and HBV-HCC. Although the principal inflammatory patterns remained largely preserved, residual confounding by liver disease severity cannot be fully excluded. Cirrhosis-restricted analyses in etiology-matched non-HCC controls were not considered sufficiently robust because only a limited number of control patients had documented cirrhosis (HDV non-HCC: n=7/17; HBV non-HCC: n=5/19). In addition, cirrhosis was not uniformly biopsy-confirmed but defined using a combination of histology, imaging, and FibroScan-based assessment, which further limits the interpretability of small subgroup analyses. Nevertheless, the principal inflammatory differences remained largely preserved after MELD adjustment across comparisons and in the cirrhosis-restricted HCC analysis, suggesting that liver disease severity alone is unlikely to fully explain the observed findings. Moreover, the present study cannot fully disentangle immune alterations attributable to chronic infection from those associated with hepatocarcinogenesis. Notably, HBV-HCC remained clinically more advanced and pro-inflammatory even after matching for tumor diameter or HBV DNA levels, raising the possibility that inflammatory pathways may contribute differently to tumor progression in HBV-HCC and HDV-HCC. Further mechanistic studies are required.

In conclusion, this study underscores the importance of consistent HCC surveillance in both HBV and HDV to enable early HCC detection and access to curative treatment options such as liver transplantation. Notably, prognosis after HCC diagnosis in HDV was not worse than in HBV. Furthermore, HDV-HCC and HBV-HCC exhibited distinct systemic inflammatory mediator profiles, indicating virus-specific systemic immune signatures associated with the underlying viral etiology. These findings provide novel insights into virus-associated systemic immune regulation in HCC and may inform future biomarker development and the investigation of immunoregulatory therapeutic strategies.

## Supplementary Material

## Data Availability

The data that support the findings of this study are available on reasonable request from Anika Wranke. Anika Wranke and Simon Glimmann had full access to all the data in the study and were responsible for the integrity and accuracy of the data analysis. Concept and design: Heiner Wedemeyer and Anika Wranke. Acquisition, analysis, and interpretation of data: Anika Wranke, Simon Glimmann, and Bernd Heinrich. Drafting of the manuscript: Anika Wranke and Bernd Heinrich. Critical revision of the manuscript for important intellectual content: All authors. Statistical analysis: Anika Wranke and Simon Glimmann. Administrative, technical, or material support: All authors. Conducting laboratory analyses: Birgit Bremer and Anika Wranke.
