# Supplementary material for: Distinct immune signatures define hepatitis D– versus hepatitis B–associated hepatocellular carcinoma
Source: Hepatol Commun. 2026 Oct 19;10(11):e1060. doi: 10.1097/HC9.0000000000001060 (PMC13682802; doi:10.1097/HC9.0000000000001060)
Supplement: Supplementary file 1 [file hc9-10-e1060-s001.docx]

**Supplementary figure legend**

Supplementary Figure 1: Significant Spearman correlations between AST and IL-8, OPG, and HGF in HBV-associated HCC.

Supplementary Fig. 2: Heat map displaying the correlation between all SIMs and clinical parameters using Spearman correlation matrix in patients with: A) HDV-HCC and B) HBV-HCC. *Correlation strength is indicated by colour intensity.*

The HDV-HCC group exhibited a preponderance of negative correlations (red dots), while the HBV-HCC group demonstrated a higher frequency of positive correlations (blue dots), indicating another SIM-profile of the two entities

**Supplementary Tables**

**Supplementary Table 1:** Plasma proteins analyzed by proximity extension assay (PEA) using the inflammation panel

| **Protein name** | **Short name** | **UniProt ID** | |
| --- | --- | --- | --- |
| C-C motif chemokine 3 | CXCL3 | P10147 |  |
| C-X-C motif chemokine 1 | CXCL1 | P09341 |  |
| Fibroblast growth factor 21 | FGF-21 | Q9NSA1 |  |
| Fibroblast growth factor 23 | FGF-23 | Q9GZV9 |  |
| Interleukin-18 | IL-18 | Q14116 |  |
| Interleukin-6 | IL-6 | P05231 |  |
| Stem cell factor | SCF | P21583 |  |
| Monocyte chemotactic protein 1 | MCP-1 | P13500 |  |
| Osteoprotegerin | OPG | O00300 |  |
| Urokinase-type plasminogen activator | uPA | P00749 |  |
| Adenosine deaminase | ADA | P00813 |  |
| Artemin | ARTN | Q5T4W7 |  |
| Axin-1 | AXIN1 | O15169 |  |
| Beta-nerve growth factor | Beta-NGF | P01138 |  |
| Brain-derived neurotrophic factor | BDNF | P23560 |  |
| Caspase 8 | CASP-8 | Q14790 |  |
| C-C motif chemokine 4 | CCL4 | P13236 |  |
| C-C motif chemokine 19 | CCL19 | Q99731 |  |
| C-C motif chemokine 20 | CCL20 | P78556 |  |
| C-C motif chemokine 23 | CCL23 | P55773 |  |
| C-C motif chemokine 25 | CCL25 | O15444 |  |
| C-C motif chemokine 28 | CCL28 | Q9NRJ3 |  |
| CD40 ligand receptor | CD40 | P25942 |  |
| CUB domain-containing protein 1 | CDCP1 | Q9H5V8 |  |
| C-X-C motif chemokine 5 | CXCL5 | P42830 |  |
| C-X-C motif chemokine 6 | CXCL6 | P80162 |  |
| C-X-C motif chemokine 9 | CXCL9 | Q07325 |  |
| C-X-C motif chemokine 10 | CXCL10 | P02778 |  |
| C-X-C motif chemokine 11 | CXCL11 | O14625 |  |
| Cystatin D | CST5 | P28325 |  |
| Delta and Notch-like EGF-related receptor | DNER | Q8NFT8 |  |
| Eotaxin-1 | CCL11 | P51671 |  |
| 4E-binding protein 1 | 4E-BP1 | Q13541 |  |
| Fibroblast growth factor 5 | FGF-5 | Q8NF90 |  |
| Fibroblast growth factor 19 | FGF-19 | O95750 |  |
| Fms-related tyrosine kinase 3 ligand | Flt3L | P49771 |  |
| Fractalkine | CX3CL1 | P78423 |  |
| Glial cell line-derived neurotrophic factor | GDNF | P39905 |  |
| Interferon gamma | IFN-gamma | P01579 |  |
| Interleukin-1 alpha | IL-1 alpha | P01583 |  |
| Interleukin-2 | IL-2 | P60568 |  |
| Interleukin-2 receptor subunit beta | IL-2RB | P14784 |  |
| Interleukin-4 | IL-4 | P05112 |  |
| Interleukin-5 | IL-5 | P05113 |  |
| Interleukin-7 | IL-7 | P13232 |  |
| Interleukin-8 | IL-8 | P10145 |  |
| Interleukin-10 | IL-10 | P22301 |  |
| Interleukin-10 receptor subunit alpha | IL-10RA | Q13651 |  |
| Interleukin-10 receptor subunit beta | IL-10RB | Q08334 |  |
| Interleukin-12 subunit beta | IL-12B | P29460 |  |
| Interleukin-13 | IL-13 | P35225 |  |
| Interleukin-15 receptor subunit alpha | IL-15RA | Q13261 |  |
| Interleukin-17A | IL-17A | Q16552 |  |
| Interleukin-17C | IL-17C | Q9P0M4 |  |
| Interleukin-18 receptor 1 | IL-18R1 | Q13478 |  |
| Interleukin-20 | IL-20 | Q9NYY1 |  |
| Interleukin-20 receptor subunit alpha | IL-20RA | Q9UHF4 |  |
| Interleukin-22 receptor subunit alpha-1 | IL-22RA1 | Q8N6P7 |  |
| Interleukin-24 | IL-24 | Q13007 |  |
| Interleukin-33 | IL-33 | O95760 |  |
| Latency-associated peptide transforming growth factor beta-1 | LAP TGF-beta-1 | P01137 |  |
| Leukemia inhibitory factor | LIF | P15018 |  |
| Leukemia inhibitory factor receptor | LIF-R | P42702 |  |
| Macrophage colony-stimulating factor 1 | CSF-1 | P09603 |  |
| Matrix metalloproteinase-1 | MMP-1 | P03956 |  |
| Matrix metalloproteinase-10 | MMP-10 | P09238 |  |
| Monocyte chemotactic protein 2 | MCP-2 | P80075 |  |
| Monocyte chemotactic protein 3 | MCP-3 | P80098 |  |
| Monocyte chemotactic protein 4 | MCP-4 | Q99616 |  |
| Natural killer cell receptor 2B4 | CD244 | Q9BZW8 |  |
| Neurotrophin-3 | NT-3 | P20783 |  |
| Neurturin | NRTN | Q99748 |  |
| Oncostatin-M | OSM | P13725 |  |
| Programmed cell death ligand 1 | PD-L1 | Q9NZQ7 |  |
| Protein S100-A12 | EN-RAGE | P80511 |  |
| Signaling lymphocytic activation molecule | SLAMF1 | Q13291 |  |
| SIR2-like protein 2 | SIRT2 | Q8IXJ6 |  |
| STAM-binding protein | STAMBP | O95630 |  |
| Sulfotransferase 1A1 | ST1A1 | P50225 |  |
| T cell surface glycoprotein CD6 | CD6 | Q8WWJ7 |  |
| T cell surface glycoprotein CD5 | CD5 | P06127 |  |
| Thymic stromal lymphopoietin | TSLP | Q969D9 |  |
| TNF-beta | TNFB | P01374 |  |
| TNF-related activation-induced cytokine | TRANCE | O14788 |  |
| Tumor necrosis factor (Ligand) superfamily member 12 | TWEAK | O43508 |  |
| Tumor necrosis factor | TNF | P01375 |  |
| Tumor necrosis factor ligand superfamily member 14 | TNFSF14 | O43557 |  |
| Tumor necrosis factor receptor superfamily member 9 | TNFRSF9 | Q07011 |  |
| Hepatocyte growth factor | HGF | P14210 |  |
| TNF-related apoptosis-inducing ligand | TRAIL | P50591 |  |
| Transforming growth factor alpha | TGF-alpha | P01135 |  |
| Vascular endothelial growth factor A | VEGF-A | P15692 |  |

**Supplementary Table 2a**. Therapies administered during follow-up in patients with chronic hepatitis D (CHD)

| HDV | 1st therapy | 2nd therapy | 3rd therapy | 4th therapy | 5th therapy | 6th therapy |
| --- | --- | --- | --- | --- | --- | --- |
| N = | 51 | 30 | 20 | 9 | 5 | 2 |
| None | 5.9 % (3) | 8.0 % (2) | 12.5 % (2) | 0 % | 20.0 % (1) | 0 |
| Resection | 17.6 % (9) | 0 % | 6.3 % (1) | 0 % | 0 | 0 |
| TACE | 33.3 % (17) | 40.0 % (10) | 25.0 % (5) | 14.3 % (1) | 20.0 % (1) | 0 |
| RFA | 5.9 % (3) | 8.0 % (2) | 12.5 % (2) | 14.3 % (1) | 20.0 % (1) | 0 |
| PEI | 7.8 % (4) | 8.0 % (2) | 0 | 14.3 % (1) | 20.0 % (1) | 0 |
| Systemic Therapy | 5.9 % (3) | 16.0 % (4) | 18.8 % (3) | 42.9 % (3) | 20.0 % (1) | 0 |
| Immunotherapy | 2.0 % (2) | 8.0 % (2) | 0 | 0 | 0 | 100 % (2) |
| Radiotherapy | 2.0 % (2) | 0 % | 0 | 0 | 0 | 0 |
| LTX | 5.9 % (3) | 16.7% (5) | 25.0 % (5) | 22.2 % (2) | 0 | 0 |
| MWA | 9.8 % (5) | 8.0 % (2) | 12.5 % (2) | 14.3 % (1) | 0 | 0 |
| TACE + Immunotherapy | 0 | 4.0 % (1) | 0 | 0 | 0 | 0 |
| TACE + PEI | 0 | 0 | 0 | 0 | 0 | 0 |
| TACE + RFA | 0 | 0 | 0 | 0 | 0 | 0 |

*Abbreviations*: TACE = Transarterial Chemoembolization; RFA = Radiofrequency Ablation; PEI = Percutaneous Ethanol Injection; LTX = Liver Transplantation; MWA = Microwave Ablation

**Supplementary Table 2b.** Therapies administered during follow-up in patients with chronic hepatitis B (CHB)

| HBV | 1st therapy | 2nd therapy | 3rd therapy | 4th therapy | 5th therapy | 6th therapy |
| --- | --- | --- | --- | --- | --- | --- |
| N = | 93 | 55 | 43 | 29 | 18 | 13 |
| None | 7.0 % (7) | 7.4 % (4) | 2.9 % (1) | 3.7 % (1) | 5.6 % (1 ) | 15.4 % (2) |
| Resection | 18.0 % (18) | 11.1 % (5) | 2.9 % (1) | 0 | 0 | 0 |
| TACE | 29.0 % (29) | 38.9 % (21) | 54.3 % (19) | 44.4 % (12) | 44.4 % (8) | 30.8 % (4) |
| RFA | 13.0 % (13) | 0 | 8.6 % (3) | 11.1 % (3) | 0 | 0 |
| PEI | 10.0% (10) | 14.8 % (8) | 8.6 % (3) | 7.4 % (2) | 16.7 % (3) | 7.7 % (1) |
| Systemic Therapy | 10.0 % (10) | 14.8 % (8) | 11.4 % (4) | 11.1 % (3) | 22.2 % (4) | 30.8 % (4) |
| Immunotherapy | 0 | 1.9 % (1) | 0 | 11.1 % (3) | 0 | 0 |
| Radiotherapy | 2.0 % (2) | 3.7 % (2) | 5.7 % (2) | 11.1 % (3) | 5.6 % (1) | 7.7 % (1) |
| LTX | 3.0 % (3) | 10.9 % (6) | 18.6% (8) | 6.9 % (2) | 0 | 0 |
| MWA | 1.0 % (1) | 0 | 2.9 % (1) | 0 | 0 | 0 |
| TACE + Immunotherapy | 0 | 0 | 0 | 0 | 0 | 0 |
| TACE + PEI | 0 | 0 | 2.9 % (1) | 0 | 0 | 0 |
| TACE RFA | 0 | 0 | 0 | 0 | 5.6 % (1) | 0 |

*Abbreviations*: HBV= Hepatitis B Virus; TACE = Transarterial Chemoembolization; RFA = Radiofrequency Ablation; PEI = Percutaneous Ethanol Injection; LTX = Liver Transplantation; MWA = Microwave Ablation

**Supplementary Table 3.** Long-term follow-up outcomes including resection, liver transplantation, and death

|  | **HDV-HCC** | **HBV-HCC** | **p-value** |
| --- | --- | --- | --- |
| Resection (%, n) | 18.5 % (10) | 22.3 % (24) | 0.585 |
| Relapse post resection (%, n) | 60.0 % (6) | 52.2 % (12) | 0.722 |
| LTX (total) (%, n) | 27.8 % (15) | 17.6 % (19) | 0.133 |
| Bridging to LTX (%, n) | 80.0 % (12) | 84.2 % (16) | 1 |
| Relapse post LTX (%, n) | 0 | 10.5 % (2) | 0.492 |
| Death (%, n) | 13.0 % (7) | 10.2 % (11) | 0.596 |

**Supplementary Table 4a:** Baseline characteristics of HCC patients with available plasma samples

|  | **HDV-HCC (n=30)** | **HBV-HCC (n=50)** | **p-value** |
| --- | --- | --- | --- |
| Age, years, mean ± SD (range) | 51.6 ± 8.7 | 60.6 ± 11.6 | <0.01 |
| Gender; male (n ,%) | 26, 86.7% | 43, 86.0% | 0.93 |
| AST (U/L),  mean±SD (range) | 96.6 ± 63 | 151.5 ± 371 | 0.22 |
| ALT (U/L),  mean±SD (range) | 73.7 ± 41 | 84.1 ± 191 | 0.39 |
| Alcaline phosphatase (U/L),  mean±SD (range) | 133.0 ± 54 | 148.4 ± 118 | 0.26 |
| Bilirubin (mg/dl), mean ± SD (range) | 44.9 ± 100 | 23.1 ± 23 | 0.13 |
| Gamma-glutamyltransferase (U/L),  mean±SD (range) | 124.4 ± 103 | 178.8 ± 220 | 0.07 |
| Cholinesterase (kU/L),  mean±SD (range) | 3.9 ± 2.2 | 5.05 ± 2.3 | 0.03 |
| Albumin (g/l), mean ± SD (range) | 29.9 ± 6.3 | 33.4 ± 6.1 | 0.05 |
| LDH (U/L),  mean±SD (range) | 242.5 ± 92 | 253.1 ± 117 | 0.36 |
| Platelets (10^3^/μL),  mean±SD (range) | 103.9 ± 46 | 155.6 ± 98 | <0.01 |
| Hemoglobin (g/dL),  mean±SD (range) | 12.6 ±2.1 | 13.0 ± 1.9 | 0.35 |
| Leucocytes (10^3^/μL), mean ± SD (range) | 4.9 ± 1.8 | 6.3 ± 2.8 | <0.01 |
| CRP (mg/l), mean±SD (range) | 6.6 ± 5.9 | 17.1 ± 33.8 | 0.04 |
| INR, mean ± SD (range) | 1.33 ± 0.4 | 1.19 ± 0.2 | 0.03 |
| AFP (U/L),  mean±SD (range) | 807.9 ± 3081 | 592.8 ± 1251 | 0.37 |
| MELD, mean ± SD (range) | 11.9 ±5.9 | 10.8 ± 3.7 | 0.15 |
| HBV DNA (IU/ml), mean ± SD (range) | 1386 ± 3690 | 782380 ± 3092183 | 0.13 |
| HDV RNA (IU/ml), mean±SD (range) | 215855.9 ± 534330.5 (600-2100000) |  |  |
| HDV RNA positive (qualitative) (%, n) | 66.7% (16/24) |  |  |
| HBeAg positive (n, %) | 5, 25% | 3, 6.5 % | 0.04 |
| Cirrhosis (n ,%) | 28, 93.3% | 38, 76.0% | 0.01 |
| Previous Antiviral therapy (n, %) | 22, 78.6% | 24, 61.5% | 0.14 |
| Previous IFN-based therapy (n, %) | 3, 10.7% | 2, 5.1% | 0.39 |
| Previous NA-based therapy (n, %) | 10, 35.7% | 22, 56.4% | 0.09 |
| Therapy at HCC diagnosis (n, %) | 25 (86%)  IFN: 1  NUCS: 24  Unknown: 1 | 35 (89%)  NUCS: 35  Unknown: 11 | 0.65 |
| ECOG (n, %)  0  1  2  3 | 19, 67.9%  5, 17.9%  3, 10.7%  1, 3.6% | 34, 85.0%  6, 15% | 0.09 |
| Previous decompensation (n, %) | 12, 50% | 10, 27% | 0.07 |
| BCLC stage (n, %)  0  A  B  C&D | 3.4% (1)  55.2% (16)  27.6% (8)  13.8% (4) | 0  46.2% (18)  33.3% (13)  20.5% (8) | 0.528 |
| Within Milan criteria (%, n) | 65.5% (19) | 50% (20) | 0.199 |
| Number of lesions (%, n)  1  2  3  4  >5 | 62.5% (15)  20.8% (5)  4.2% (1)  8.3% (2)  4.2% (1) | 51.4% (18)  34.3% (12)  5.7% (2)  2.9% (1)  5.7% (2) | 0.703 |
| Largest lesion diameter (mm),  mean±SD (range) | 35.7 ± 23.3 (8-95) | 48.7 mm ± 35.9 (10-170) | 0.117 |
| Suspicious lymph nodes (%, n) | 16% (4) | 37.1 % (13) | 0.065 |
| Portal vein invasion (%, n) | 4.0% (1) | 13.9% (5) | 0.205 |
| Satellite lesions (%, n) | 0 | 8.1% (3) | 0.205 |
| Extrahepatic lesions (%, n) | 0 | 6.8% (4) | 0.102 |

**Supplementary Table 4b.** Baseline characteristics of HDV-associated HCC patients with available plasma samples compared with HDV control patients

|  | **HDV-HCC (n=30)** | **HDV-control**  **(n=17)** | **p-value** |
| --- | --- | --- | --- |
| Age, years, mean ± SD (range) | 51.6 ± 8.7 | 51.1 ± 10.9 | 0.85 |
| Gender; male (n ,%) | 26, 86.7% | 14, 82.4% | 0.69 |
| AST (U/L), mean±SD (range) | 96.6 ± 63 | 126.5 ±255 | 0.56 |
| ALT (U/L), mean±SD (range) | 73.7 ± 41 | 141.8 ± 284 | 0.34 |
| Alcaline phosphatase (U/L),  mean±SD (range) | 133.0 ± 54 | 94.2 ±31.5 | <0.01 |
| Bilirubin (mg/dl), mean ± SD (range) | 44.9 ± 100 | 44.8 ±113 | 0.99 |
| Gamma-glutamyltransferase (U/L), mean±SD (range) | 124.4 ± 103 | 50.3 ±40.8 | <0.01 |
| Cholinesterase (kU/L),  mean±SD (range) | 3.9 ± 2.2 | 6.8 ± 2.1 | <0.01 |
| Albumin (g/l), mean ± SD (range) | 29.9 ± 6.3 | 38.8 ± 4.5 | <0.01 |
| Platelets (10^3^/μL), mean±SD (range) | 103.9 ± 46 | 132.1 ± 71.2 | 0.15 |
| Hemoglobin (g/dL), mean±SD (range) | 12.6 ± 2.0 | 13.4 ±1.3 | 0.17 |
| Leucocytes (10^3^/μL), mean ± SD (range) | 4.9 ± 1.8 | 4.9 ± 2.1 | 0.98 |
| CRP (mg/l), mean±SD (range) | 6.6 ± 5.9 | 4.7 ± 5.8 | 0.34 |
| INR, mean ± SD (range) | 1.33 ± 0.4 | 1.25 ±0.4 | 0.56 |
| AFP (U/L), mean±SD (range) | 807.9 ± 3081 | 7.4 ± 6.1 | 0.36 |
| MELD, mean ± SD (range) | 11.9 ±5.9 | 10.0 ± 4.6 | 0.27 |
| HBV DNA (IU/ml), mean ± SD (range) | 1386 ± 3690 | 14769 ±43394 | 0.31 |
| HDV RNA ± SD | 140775.6 ±439018 | 234057.7 ±454012 | 0.65 |
| HBeAg positive (n, %) | 5, 25% | 5, 29.4% | 0.76 |
| Cirrhosis (n ,%) | 27, 100% | 7, 41.2% | <0.01 |
| Previous Antiviral therapy (n, %) | 22, 78.6% | 2, 11.8% | <0.01 |
| Previous IFN-based therapy (n, %) | 3, 10.7% | 1, 5.9 % | 0.58 |
| Previous NA-based therapy (n, %) | 10, 35.7% | 1, 5,9% | 0.02 |

**Supplementary Table 4c.** Baseline characteristics of HBV-associated HCC patients with available plasma samples compared with HBV control patients

|  | **HBV-HCC (n=50)** | **HBV-control (n=19)** | **p-value** |
| --- | --- | --- | --- |
| Age, years, mean ± SD (range) | 60.6 ± 11.6 | 55.4 ± 8.6 | 0.08 |
| Gender; male (n ,%) | 43, 86.0% | 13, 68.4% | 0.09 |
| AST (U/L),  mean±SD (range) | 151.5 ± 371 | 128.2 ± 316 | 0.81 |
| ALT (U/L),  mean±SD (range) | 84.1 ± 191 | 109.5 ± 165 | 0.62 |
| Alcaline phosphatase (U/L),  mean±SD (range) | 148.4 ± 118 | 104.4 ± 68 | 0.14 |
| Bilirubin (mg/dl), mean ± SD (range) | 23.1 ± 23 | 29.9 ± 77 | 0.16 |
| Gamma-glutamyltransferase (U/L), mean±SD (range) | 178.8 ± 220 | 115.8 ± 225 | 0.31 |
| Cholinesterase (kU/L),  mean±SD (range) | 5.05 ± 2.3 | 8.0 ± 2.9 | <0.01 |
| Albumin (g/l), mean ± SD (range) | 33.4 ± 6.1 | 38.6 ± 6.2 | 0.01 |
| Platelets (10^3^/μL),  mean±SD (range) | 155.6 ± 98 | 203.6 ± 86 | 0.07 |
| Hemoglobin (g/dL),  mean±SD (range) | 13.0 ± 1.9 | 13.9 ±1.8 | 0.12 |
| Leucocytes (10^3^/μL), mean ± SD (range) | 6.3 ± 2.8 | 5.9 ± 1.4 | 0.63 |
| CRP (mg/l), mean±SD (range) | 17.1 ± 33.8 | 3.8 ± 7.9 | 0.01 |
| INR, mean ± SD (range) | 1.19 ± 0.2 | 1.17 ± 0.2 | 0.10 |
| AFP (U/L), mean±SD (range) | 592.8 ± 1251 | 8.4 ± 7.4 | 0.01 |
| MELD, mean ± SD (range) | 10.8 ± 3.7 | 8.8 ±3.0 | 0.04 |
| HBV DNA (IU/ml), mean ± SD (range) | 782380 ± 3092183 | 94123125 ± 239629931 | 0.14 |
| HBeAg positive (n, %) | 3, 6.5 % | 4, 21.1% | 0.09 |
| Cirrhosis (n ,%) | 38, 79.2% | 5, 26.3% | <0.01 |
| Previous Antiviral therapy (n, %) | 24, 61.5% | 13, 72.2 % | 0.43 |
| Previous IFN-based therapy (n, %) | 2, 5.1% | 6, 33.3% | <0.01 |
| Previous NA alone therapy (n, %) | 22, 56.4% | 7, 38.9% | 0.22 |

**Supplementary Table 4d.** Baseline characteristics of control patients with available plasma samples

|  | **HDV-control (n=17)** | **HBV-control (n=19)** | **p-value** |
| --- | --- | --- | --- |
| Age, years, mean ± SD (range) | 51.1 ± 10.9 | 55.4 ± 8.6 | 0.19 |
| Gender; male (n ,%) | 14, 82.4% | 13, 68.4% | 0.34 |
| AST (U/L),  mean±SD (range) | 126.5 ±255 | 128.2 ± 316 | 0.97 |
| ALT (U/L),  mean±SD (range) | 141.8 ± 284 | 109.5 ± 165 | 0.68 |
| Alcaline phosphatase (U/L),  mean±SD (range) | 94.2 ±31.5 | 104.4 ± 68 | 0.58 |
| Bilirubin (mg/dl), mean ± SD (range) | 44.8 ±113 | 29.9 ± 77 | 0.64 |
| Gamma-glutamyltransferase (U/L), mean±SD (range) | 50.3 ±40.8 | 115.8 ± 225 | 0.24 |
| Cholinesterase (kU/L),  mean±SD (range) | 6.8 ± 2.1 | 8.0 ± 2.9 | 0.15 |
| Albumin (g/l), mean ± SD (range) | 38.8 ± 4.5 | 38.6 ± 6.2 | 0.92 |
| Platelets (10^3^/μL),  mean±SD (range) | 132.1 ± 71.2 | 203.6 ± 86 | 0.01 |
| Hemoglobin (g/dL),  mean±SD (range) | 13.4 ±1.3 | 13.9 ±1.8 | 0.35 |
| Leucocytes (10^3^/μL), mean ± SD (range) | 4.9 ± 2.1 | 5.9 ± 1.4 | 0.11 |
| CRP (mg/l), mean±SD (range) | 4.7 ± 5.8 | 3.8 ± 7.9 | 0.74 |
| INR, mean ± SD (range) | 1.25 ±0.4 | 1.17 ± 0.2 | 0.20 |
| AFP (U/L), mean±SD (range) | 7.4 ± 6.1 | 8.4 ± 7.4 | 0.73 |
| MELD, mean ± SD (range) | 10.0 ± 4.6 | 8.8 ±3.0 | 0.33 |
| HBV DNA (IU/ml), mean ± SD (range) | 14769 ±43394 | 94123125 ± 239629931 | 0.14 |
| HBeAg positive (n, %) | 5, 29.4% | 4, 21.1% | 0.56 |
| Cirrhosis (n ,%) | 7, 41.2% | 5, 26.3% | 0.35 |
| Previous Antiviral therapy (n, %) | 2, 11.8% | 13, 72.2 % | <0,01 |
| Previous IFN-based therapy (n, %) | 1, 5.9 % | 6, 33.3% | 0.05 |
| Previous NUCs alone therapy (n, %) | 1, 5,9% | 7, 38.9% | 0.02 |
| Therapy at time of serology | 13 (72%)  IFN:5 (29.4%)  NUCS: 8 (47.1%) | 18 (95%)  NUCS: 18 (94.7%) | 0.05 |

**Supplementary Table 5.** Sensitivity analysis restricted to cirrhotic HCC patients (HDV-HCC vs. HBV-HCC)

| **SIM** | **Higher in main analysis** | **Higher in cirrhosis-only analysis** | **p value (cirrhosis only)** | **Direction preserved** | **Comment** |
| --- | --- | --- | --- | --- | --- |
| CD244 | HDV-HCC | HDV-HCC | 0.002 | Yes | Main finding preserved |
| CD6 | HDV-HCC | HDV-HCC | <0.001 | Yes | Main finding preserved |
| DNER | HDV-HCC | HDV-HCC | 0.002 | Yes | Main finding preserved |
| IL10 | HDV-HCC | HDV-HCC | 0.003 | Yes | Main finding preserved |
| SLAMF1 | HDV-HCC | HDV-HCC | 0.008 | Yes | Main finding preserved |
| uPA | HDV-HCC | HDV-HCC | 0.006 | Yes | Main finding preserved |
| FGF21 | HBV-HCC | HBV-HCC | 0.010 | Yes | Main finding preserved |
| SCF | HBV-HCC | HBV-HCC | <0.001 | Yes | Main finding preserved |
| LIF | HBV-HCC | HBV-HCC | 0.008 | Yes | Main finding preserved |
| VEGFA | HBV-HCC | HBV-HCC | 0.008 | Yes | Main finding preserved |
| OSM | HBV-HCC | HBV-HCC | 0.008 | Yes | Main finding preserved |
| TNFβ | HDV-HCC | HDV-HCC | 0.014 | Yes | Main finding preserved |
| CDCP1 | HDV-HCC | HDV-HCC | 0.030 | Yes | Main finding preserved |
| LIFR | HDV-HCC | HDV-HCC | 0.039 | Yes | Main finding preserved |
| IL2 | HBV-HCC | HBV-HCC | 0.020 | Yes | Main finding preserved |
| EN-RAGE | HBV-HCC | HBV-HCC | 0.040 | Yes | Main finding preserved |
| FGF5 | HBV-HCC | HBV-HCC | 0.035 | Yes | Main finding preserved |
| IL20RA | HBV-HCC | HBV-HCC | 0.007 | Yes | Main finding preserved |
| CXCL5 | HBV-HCC | HBV-HCC | 0.010 | Yes | Main finding preserved |
| CXCL10 | — | HDV-HCC | 0.030 | — | Additional finding (exploratory) |
| CXCL11 | — | HDV-HCC | 0.030 | — | Additional finding (exploratory) |
| IL2RB | — | HBV-HCC | 0.025 | — | Additional finding (exploratory) |
| TRAIL | — | HDV-HCC | 0.038 | — | Additional finding (exploratory) |
| BetaNGF | — | HBV-HCC | 0.025 | — | Additional finding (exploratory) |

Footnote: Sensitivity analysis restricted to cirrhotic HCC patients (HDV-HCC n = 28; HBV-HCC n = 38). One-sided p values are shown to maintain consistency with the primary analyses. “Main finding preserved” indicates mediators that remained significant and directionally concordant with the primary analysis. “Additional finding (exploratory)” denotes mediators identified only in the cirrhosis-restricted analysis and not in the primary comparison.
